# Quercetin inhibits angiotensin II-induced vascular smooth muscle cell proliferation and activation of JAK2/STAT3 pathway: A target based networking pharmacology approach

**DOI:** 10.3389/fphar.2022.1002363

**Published:** 2022-10-17

**Authors:** Di Wang, Farman Ali, Huixin Liu, Ying Cheng, Meizhu Wu, Muhammad Zubair Saleem, Huifang Zheng, Lihui Wei, Jiangfeng Chu, Qiurong Xie, Aling Shen, Jun Peng

**Affiliations:** ^1^ Clinical Research Institute, the Second Affiliated Hospital and Academy of Integrative Medicine, Fujian University of Traditional Chinese Medicine, Fuzhou, Fujian, China; ^2^ Fujian Key Laboratory of Integrative Medicine on Geriatrics, Fujian University of Traditional Chinese Medicine, Fuzhou, Fujian, China; ^3^ Fujian Collaborative Innovation Center for Integrative Medicine in Prevention and Treatment of Major Chronic Cardiovascular Diseases, Fuzhou, Fujian, China; ^4^ Fujian Key Laboratory of Natural Medicine Pharmacology, Fujian Medical University, Fuzhou, Fujian, China

**Keywords:** networking pharmacology, JAK2, quercetin, vascular smooth muscle cells, stat3

## Abstract

The rapid growth of vascular smooth muscle cells (VSMCs) represents crucial pathological changes during the development of hypertensive vascular remodeling. Although quercetin exhibits significantly therapeutic effects on antihypertension, the systematic role of quercetin and its exact mode of action in relation to the VSMCs growth and its hypertension-related networking pharmacology is not well-documented. Therefore, the effect of quercetin was investigated using networking pharmacology followed by *in vitro* strategies to explore its efficacy against angiotensin II (Ang II)-induced cell proliferation. Putative genes of hypertension and quercetin were collected using database mining, and their correlation was investigated. Subsequently, a network of protein-protein interactions was constructed and gene ontology (GO) analysis was performed to identify the role of important genes (including CCND1) and key signaling pathways [including cell proliferation and Janus kinase 2/signal transducer and activator of transcription 3 (JAK2/STAT3) pathway]. We therefore further investigated the effects of quercetin in Ang II-stimulated VSMCs. This current research revealed that quercetin significantly reduced the cell confluency, cell number, and cell viability, as well as expression of proliferating cell nuclear antigen (PCNA) in Ang II-stimulated VSMCs. Mechanistic study by western blotting confirmed that quercetin treatment attenuated the activation of JAK2 and STAT3 by reducing its phosphorylation in Ang II stimulated VSMCs. Collectively, the current study revealed the inhibitory effects of quercetin on proliferation of Ang II stimulated VSMCs, by inhibiting the activation of JAK2/STAT3 signaling might be one of underlying mechanisms.

## Introduction

High blood pressure is a key risk factor for cardiovascular disorders. It is one of the major causes of heart attack that affects more than one billion people globally (Vos et al., 2020). The steadily rise of blood pressure leads to renal disease, myocardial infarction, and heart related disorders (Ku et al., 2019; Lv and Zhang, 2019). Essential hypertension (EH) is a disease associated with increased systemic circulatory arterial blood pressure induced by an interplay between genetic and environmental factors (Natekar et al., 2014; Sanidas et al., 2017). EH affects 95% of hypertensive patients without knowing the causative factors, which mostly involve sympathetic nervous system hyperactivity, renal mechanisms, dysfunction of endothelial cells, nitric oxide pathway, enhanced left ventricular ejection force, and high blood pressure (Boutouyrie et al., 2021).

According to the American Society for Hypertension, lowering the blood pressure should not only be the main objective but EH could be treated with medications to prevent the impending cardiovascular syndrome (Beaney et al., 2018; Mancusi et al., 2018). Antihypertensive drugs currently used in clinics to treat hypertension are divided into five classes. These include calcium channel blockers, angiotensin-converting enzyme (ACE) inhibitors, thiazide diuretics, angiotensin II (Ang II) receptor blockers and beta blockers (Kim et al., 2019; Brouwers et al., 2021). However, some of antihypertensive drugs exist several limitation and side effects, such as, thiazide-like diuretic causes hyperuricemia and hyperlipidemia. ACE inhibitors has several side effects, e.g., cough, hypotension, fatigue, and azotemia (Parati et al., 2021; Khalil and Zeltser, 2022). EH remains an incurable illness, therefore new effective treatment strategies remain to be further explored (Tsang et al., 2020; Jama et al., 2022).

Discovery of new antihypertensive natural compounds with less side effects is an urgent need for the treatment of hypertension. Thus, flavonoid molecules, like polyphenols, and flavanols are essential antioxidant sources in the nutrition (Anwar et al., 2019). Flavonoid consumption has been linked to lower the cardiovascular-associated mortality (Clark et al., 2015; Gee and Ahluwalia, 2016). Quercetin is a flavonoid with potential antioxidant, antiviral, anti-inflammatory, anticancer and vasodilating properties (Guillermo Gormaz et al., 2015; Griffiths et al., 2016). Quercetin decreased the oxidative stress, elevated nitric oxide biosynthesis, and minimized the dysfunction of arterial endothelium cells (Pereira et al., 2018). Quercetin inhibits adipogenesis through activation of AMP-activated protein kinase (AMPK) pathway and it also causes apoptosis in mature adipocytes by inhibiting extracellular signal-regulated kinase 1/2 (ERK1/2) and c-Jun N-terminal kinase (JNK) phosphorylation and activating the apoptosis pathway (Zhao et al., 2021). Quercetin plays anti-inflammatory role by supression of the nuclear factor kappa B (NF-κB) pathway (Comalada et al., 2005; Cheng et al., 2019). Thus, quercetin has the potential to be a leading molecule in drug discovery research (Patel et al., 2018). Some previous literature has revealed that quercetin attenuates the activation of signaling pathways, that further leads to reduce vascular injury and inflammation (Almatroodi et al., 2021). Quercetin has antihypertensive properties by improving endothelial dysfunction, lowering blood pressure *via* inhibition of potassium channel activity, and regulating the functions of several signaling pathways (Xue et al., 2018; Elbarbry et al., 2020). However, the underlying molecular mechanism of quercetin on antihypertension remains largely unknown.

The networking pharmacology focuses on the interactions between drugs and their targets and point out the way for the discovery of novel drug molecules. This strategy is particularly suitable for the investigation of complicated illnesses like EH (Anighoro et al., 2014). Therefore, our results identified the direct and indirect genes (GJA1, PPARG, JAK2, and STAT3) associated with quercetin. These targets were enriched into several GO processes in the development of the circulatory system, regulation of cell proliferation, blood vessel development, and also enriched in numerous Kyoto Encyclopedia of Genes and Genomes database (KEGG) pathways including JAK/STAT pathway. Further, we found that Ang II stimulates the proliferation of VSMCs and quercetin treatment reduces the expression of the proliferative biomarker PCNA. Moreover, Ang II leads to the proliferation of VSMCs while quercetin treatment reduces proliferative capabilities of VSMCs and inhibits JAK2/STAT3 activation.

## Materials and methods

Networking pharmacology was employed to identify the quercetin mechanism for the treatment of hypertension. Traditional Chinese Medicine Systems Pharmacology Database (TCMSP), Binding database (BDB) and STITCH databases (version 5.0) were used to screen the targets of quercetin. The disease targets were screened through CooLGeN, MalaCards, Therapeutic Target Database (TTD) and Online Mendelian Inheritance in Man (OMIM) databases. The relationship between the “cross gene of quercetin and EH” and the phenotype of “essential hypertension” was studied with VarElect, and the common targets were acquired by mapping with the drug action targets. GeneMANIA database is used for research to generate cross targets of quercetin and EH and protein interaction network of corresponding genes, and key targets are screened according to the size of interaction relationship. The “quercetin-potential target” network diagram was drawn by using the Cytoscape 3.7.2 software. Using Database for Annotation, Visualization, and Integrated Discovery (DAVID) database, the GO function and KEGG pathway enrichment of the targets were analyzed. Finally, *in vitro* studies were conducted to explore the function of quercetin on anti-proliferation and the activation of JAK2/STAT3 signaling pathway in Ang II stimulated VSMCs.

### Analysis of drug likeness

As per the PubChem database, a wide range of physical and chemical characteristics of quercetin was determined depending on Lipinski’s rule of five (RO5). Following factors were considered for further characterization of quercetin; molecular weight or mass (MW) number of H-bond donor and acceptor, number of rotatable bonds, TPSA (topological polar surface area), and octanol-water partition coefficient (XLogP3). Swiss ADMET as well as the ADMET descriptor in Discovery Studio 2016 were used to examine the absorption characteristics of quercetin, namely the blood brain barrier (BBB) and human gastrointestinal absorption (HGIA) plots for clear understanding of the ADMET characteristics of quercetin (Daina et al., 2017; Vora et al., 2019).

### Prediction of quercetin targets

To identify the targets of quercetin, researchers used a number of strategies relying mostly on structural similarities principle, reversible docking technique, and data integration as well as assimilation process. PharmMapper Server, BDB, TCMSP, Swiss Target Prediction, and STITCH database were used to compile the potential targets (Ru et al., 2014; Daina and Zoete, 2016; Wang et al., 2017; Daina et al., 2019). The name of the compound quercetin was used to acquire target genes from STITCH and TCMSP databases. We acquired anticipated genes by SMILES string of quercetin in the Swiss Target Prediction and BDB database focused on the structure resemblance criteria. Furthermore, the structure of quercetin in SDF file format (PubChem CID: 5280343) were uploaded into PharmMapper for potential gene identification, followed by the normalization of acquired genes performed by using the UniProt Database. Lastly, these obtained genes were integrated, after exclusion of redundant targets (Supplementary Table S2). This section of the gene set is referred to as “quercetin 182 targets” within subsequent summary.

### Acquiring the essential hypertension-related targets

EH-linked genes were obtained from the CooLGeN database Drug Bank database, MalaCards database, Therapeutic Target Database (TTD), and OMIM database, to determine the accuracy of disease-related genes. The scores of targets relatively greater than genes were extracted from the CooLGeN database. EH-associated targets were retrieved the OMIM and MalaCards databases. Redundant genes were removed from the datasets, and total 483 EH-associated targets were chosen for further study. This section of the gene array is referred to as “genes associated with EH.” Supplementary Table S2 provides more detailed information for the targets. The correlation within these two classes of targets could be one of the potential genes of quercetin. The above category of integrating targets is referred to as “intersection genes of quercetin and essential hypertension”.

### Quercetin targets including essential hypertension phenotypic associations assessment

The VarElect database leads to more accurate identification of genes that are often directly or indirectly linked to a specific phenotype or disease (Stelzer et al., 2016). VarElect’s ability to interpret and score gene lists dependent on phenotype keywords inputted is a critical feature. Elastic search computing is largely responsible for the VarElect analysis scores. The intensity of a term’s occurrence in particular GeneCards is relative to the rate *via* its expression within these genes (opposite report intensity rectification) to evaluate its score. The association between “the intersection genes of quercetin as well as EH” and also the phenotype of “Essential hypertension” was investigated using the VarElect.

### Evaluation of protein-protein interactions

In addition to creating PPI networks, the GeneMANIA platform can also find a set of targets similar to those entered. It is based on a variety of functionally relevant information, and compared differences between them, i.e., co-expression as well as co-localization, using a variety of analogous information (Ogris et al., 2018). The GeneMANIA database was used in research to generate protein-protein interaction (PPI) network of intersecting targets of quercetin and EH along with the corresponding genes. We may obtain not only the association between the entered intersecting targets, but also many other target genes that are tightly associated to these targets, using GeneMANIA examination. The above unique set of genes is termed “anticipated targets of quercetin anti-EH” within current analysis. The Network Analyzer tool in Cytoscape 3.7.2 was used to calculate topological characteristics of the PPI network, such as degree, closeness centrality, betweenness centrality, and average shortest path length.

### Enrichment of gene ontology evaluation

Metascape is an effective gene annotation and enriched analysis program (Zhou et al., 2019). The predicted quercetin anti-EH targets were identified and categorized into various GO pathways. To obtain the biological processes as well as KEGG pathways relevant to the mechanism of EH, DAVID (version 6.8) tool was used.

### Implementing a target-pathway/function network

The network was created using Cytoscape 3.7.2 for illustration of complicated networks. The nodes of the network identified potential quercetin targets for EH, biological processes, and signaling pathways discovered by enrichment analysis, and the edges represented associations between them.

### Chemicals/reagents

Quercetin (Cat.no.Q4951-10G) was purchased from Sigma (St.Louis, MO, United States) with >95% purity (HPLC). The protein assay reagent kit bicinchoninic acid (BCA), trypsin-EDTA, fetal bovine serum (FBS) and Dulbecco’s Modified Eagle Medium (DMEM) were obtained from Thermo Fisher Scientific (Waltham, MA United States). Phosphate saline buffer was purchased from Maixin Biotechnology (Fuzhou, Fujian China), and polyvinylidene fluoride (PVDF) membrane was purchased from Millipore (Billireca, MA, United States). Antibodies against STAT3 (Cat no.41464), p-JAK2 (Cat no.11151) were purchased from Signaling antibody (SAB) (College Park, Maryland, United States). The α-SMA (Cat no.19245S), p-STAT3 (Cat no.9145S), JAK2 (Cat no. 3032S) antibodies were purchased from Cell Signaling Technology (Danvers, MA, United States). PCNA (Cat no. ab29), goat polyclonal secondary antibody to rabbit IgG H&L (Alexa Fluor^®^ 647; Cat no. ab150079), Ang II (Cat no. ab120183) were purchased from Abcam (Cambridge Science Park, Cambridge, United Kingdom) and anti-rabbit secondary antibody (Cat no. 7074) was purchased from Cell Signaling Technology (Danvers, MA, United States). Antibody against GAPDH (Cat no. Abp57259) and kit of CCK-8 (Cat no. ktc011001) were obtained from Abbkine (Wuhan, Hubei, China).

### Vascular smooth muscle cells isolation

Wistar rats were anesthetized by using isoflurane; abdominal aorta was instantaneously dissected, washed with ice-chilled 1.5 mM of CaCl_2_-HEPES-buffered salt solution (HBSS) for the removal of blood. After that, the aorta was cut longitudinally and the endothelial cells were removed with a cotton swab retrieved delicately. Cells were kept in 1.5 mM CaCl_2_-HBSS at 4°C for 30 min and were treated with Ca^2+^ free-HBSS at room temperature for 20 min. Then, a digestion solution containing mixed enzymes (0.5 ml), collagenase (3.2 mg), BSA (2 mg), and papain (0.3 mg) was prepared, and the aorta was digested at 37°C for 20–30 min. After the removal of extra enzyme, Ca^2+^ free-HBSS was used to wash the cells and kept in T25 flask with 5 ml of 10% FBS containing DMEM medium supplemented with 100 μg/ml streptomycin and 100 IU/ml penicillin. Lastly, by pipetting up and down for 50 to 60 times, gently dispersed VSMCs were kept in incubator containing 5% CO_2_. For each experiment fresh VSMCs were cultured and used for the corresponding experiments at third or fourth passage. All animal experiment protocols were approved by the Animal Ethics Committee of Fujian University of Traditional Chinese Medicine (No. FJTCM IACUC. 2021185).

### Immunofluorescence analysis

Isolated primary VSMCs were seeded into glass bottom dishes at a density of 0.8 × 10^5^ cells/well. Following 24 h of cultivation, cells were permeabilized with 0.25% Triton X-100 for 10 min, fixed with 4% paraformaldehyde for 15 min, and then blocked with blocking buffer (containing of 5% BSA, 85% PBS and 10% goat serum) for 1 h. Subsequently, cells were incubated with antibody against α-SMA (1:200) at 4°C overnight, followed by washing with PBS for thrice and incubated with secondary antibody anti-rabbit (1:400) in the dark at room temperature for 1 h. Then, cells were washed thrice with PBS and incubated with Hoechst stain (Thermo Fisher Scientific) for 10 min and observed under confocal microscope (PerkinElmer Inc., Waltham, MA, United States).

### Cell counting Kit-8 assay for cell viability analysis

The cell viability assay was determined using cell counting assay. The extracted VSMCs in suspension at 3 × 10^4^ cells/mL density was seeded into 96 well plates. When VSMCs confluency was reached to 30%–40%, cells were placed in serum free media for starvation for 6–8 h and after that VSMCs were treated with various concentrations of quercetin (3.125, 6.25,12.5, 25, 50, and 100 μM) for 24 h or Ang II (0.01, 0.1, 1, and 10 μM) for 24 h. Besides, 0.1 μM of Ang II stimulated VSMC_S_ were treated with quercetin (6.25, 12.5, and 25 μM) for 24 h. Following treatment, each well loaded with 10 µl of the CCK8 solution, which was then incubated at 37°C for 2 h. By using micro plate reader (Multiskan FC, Thermo Fisher Scientific) absorbance was measured at 450 nm.

### Western blotting analysis

For the extraction of total proteins, Western cell lysis buffer (Beyotime Biotechnology, Shanghai, China) supplemented with 1 mM phenylmethylsulfonyl fluoride (PMSF) and other protease inhibitors including cocktail (MedChemExpress, Monmouth Junction, NJ, United States), and phosStop (Roche; Basel, Switzerland) was used. Briefly, cells were lysed with the lysis buffer for 20 min in ice and centrifuged at 14,000 g at 4°C for 20 min as described previously (Liu et al., 2021). Followed by the collection of supernatants, total concentration of protein was assessed by BCA protein kit assay and equal amount of each sample were subjected for separation through 10% sodium dodecyl sulfate polyacrylamide gel electrophoresis (SDS-PAGE). Proteins were transferred to PVDF membranes using wet transfer system. Further, 5% skimmed milk was used for the blockage of membranes for 2 h at room temperature and then primary antibodies; anti-p-JAK2, anti-JAK2, anti-p-STAT3, anti-STAT3, anti-PCNA or anti-GAPDH (dilution factor 1:1000) were incubated at 4°C for overnight. After that, membranes were washed thrice with TBST buffer and membranes were incubated with anti-rabbit or mouse secondary antibody conjugated to horse radish peroxidase (dilution factor 1:5000) for 1 h at room temperature. The protein expression was detected by chemiluminescence kit and ImageJ Software.

### Statistical analyses

Statistical Package for the Social Sciences (SPSS 25.0) software (Chicago, IL, United States) was used for statistical analysis. All data were provided as means ± SD the experiment. The Shapiro-Wilk test was employed to determine normality in experiments with three or more groups. When the data meet a normal distribution, one-way analysis of variance (ANOVA) was used, followed by the Bonferroni post-hoc test for pairwise comparisons. The non-parametric Kruskal–Wallis test was used to analyze the non-normally distributed data, pursued by the Dunnett’s *t*-test for paired comparisons. The significant difference was considered with *p < 0.05*.

## Results

### An evaluation of Quercetin’s drug likeness

For health impact assessment (HIA) and blood brain barrier (BBB), analysis of absorption, distribution, metabolism, excretion, and toxicity (ADMET) provides four levels of prediction between 95% and 99% of ellipsoids confidence. Quercetin was well absorbed *via* intestine and also had very high BBB level of penetration, as seen in Table 1. Supplementary Figure S1A illustrates the chemical structure, while Supplementary Figure S1B demonstrates the suitable physicochemical space for oral bioavailability (OB) as well as pharmacokinetic characteristics including TPSA 131.36Å, nine rotatable bonds, and so on. In addition, the BOILED-EGG analysis revealed that quercetin penetration into the blood-brain [−1.098 (log BB)] and 77.207% absorption in the human gastrointestinal tract was illustrated in orange and colored zones respectively, as given in Supplementary Figure S1C plot.

**TABLE 1:** Quercetin’s molecular and physicochemical properties.

Properties	Parameters
MW	302.24 g/mol
hydrogen bond donor	5
hydrogen bond acceptors	7
lipophilicity (logP)	0.524260
XLogP3	1.53
No. Rotatable bonds	9
Molar refractivity	64.369995
TPSA	131.36 Å
GIT absorption	77.207%
Level of BBB	−1.098 (log BB)

MW, Molecular weight; TPSA, Topological polar surface area; BBB, blood brain barrier; GIT, gastrointestinal; XLogP3, water partition coefficient/computed octanol.

### Quercetin targets and essential hypertension correlation analysis

**FIGURE 1:**
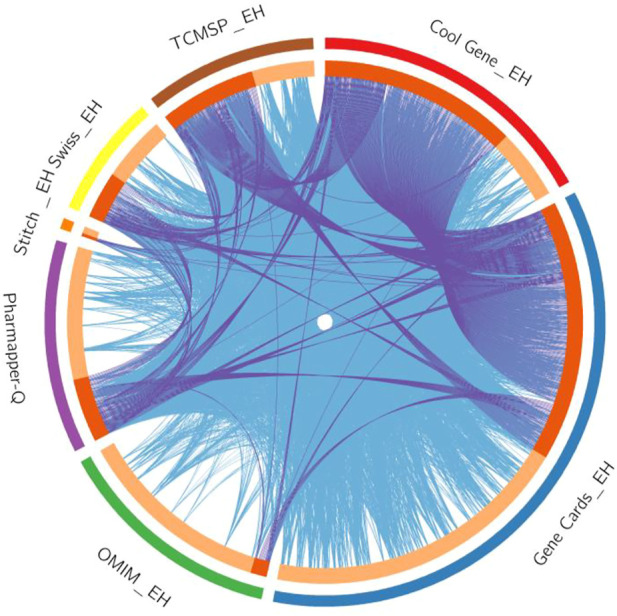
The Circos plot shows intersecting genes. The outer arcs symbolize the identity of each gene. Each of the inside arcs represents a gene, and each gene is represented by its own spot on the arc. The dark orange color indicates genes found in multiple databases, whereas the light orange color signifies genes found only in that gene datasets. Purple lines connect identical gene that seems in specific genes databases. Blue lines connect the genes that identified as similar ontologies.

The intersection of targets from various databases was depicted by Circos plot (Figure 1). To identify gene overlaps, relatively similar enriched ontology term(s) and their functions or shared pathways were used to significantly improve gene overlaps as given in Supplementary Table S1. To study the genotype-phenotype correlations, a number of 54 overlapping genes of quercetin and EH were entered into the VarElect interactive platform. The results are presented in Table 2. The GJA1 gene on chromosome six encodes the gap junction alpha-1 protein (GJA1), widely recognized as connexin 43 (Cx43). Connexin (Cx) protein isoforms give a shape to gap junctions (GJs), which acts as a pathway for information exchange between cells and expressed in blood vessels in four different forms: Cx37, Cx40, Cx43, and Cx45. MAPK1 commonly recognized as p42MAPK and ERK2 is a mitogen-activated protein kinase enzyme that is denoted via MAPK1 gene in humans. MAP Kinases are involved in several cellular activities, like differentiation, proliferation, expression control, and growth, as well as acting as an integrating site for various biochemical signals. STAT3 is an essential intercellular signaling circuit and acts on several downstream genes such as CyclinD1, c-myc, and VEGFs. Activation of STAT3 depends on stimulation of the signal transduction protein JAK2 by various cytokines such as interleukins and growth factors (HGF and EGF) and results in phosphorylation of STAT3 at 705 tyrosine amino acid. STAT3, which is continuously activated, promotes tumor cell proliferation and survival, stimulates angiogenesis, and inhibits the immune response to malignancy.

**TABLE 2:** Analysis of Quercetin targets with the phenotype “Essential hypertension” using VarElect.

S.No	Description	Gene symbols	Score	Phenotypic correlations
1	Gap Junction Protein Alpha 1	GJA1	31.76	Directly
2	Caveolin 1	CAV1	31.41	Directly
3	Peroxisome Proliferator Activated Receptor Gamma	PPARG	30.41	Directly
4	Fos Proto-Oncogene, AP-1 Transcription Factor Subunit	FOS	25.46	Directly
5	Collagen Type III Alpha 1 Chain	COL3A1	23.69	Directly
6	Raf-1 Proto-Oncogene, Serine/Threonine Kinase	RAF1	23.57	Directly
7	Cyclin D1	CCND1	18.43	Directly
8	Phosphatase And Tensin Homolog	PTEN	16.30	Directly
9	Transforming Growth Factor Beta 1	TGFB1	16.19	Directly
10	Bone Morphogenetic Protein 2	BMP2	15.66	Directly
11	Interleukin 10	IL10	14.73	Directly
12	Matrix Metallopeptidase 1	MMP1	11.78	Directly
13	Adrenoceptor Beta 2	ADRB2	11.36	Directly
14	Nitric Oxide Synthase 3	NOS3	10.52	Directly
15	Insulin Like Growth Factor 2	IGF2	9.59	Directly
16	Tumor Protein P53	TP53	6.29	Directly
17	Thrombomodulin	THBD	6.29	Directly
18	Heme Oxygenase 1	HMOX1	4.47	Directly
19	Mitogen-Activated Protein Kinase 1	MAPK1	3.44	Directly
20	Secreted Phosphoprotein 1	SPP1	2.43	Directly
21	Interleukin 6	IL6	2.43	Directly
22	Cyclin Dependent Kinase Inhibitor 2A	CDKN2A	1.62	Directly
23	Interleukin 1 Beta	IL1B	1.42	Directly
24	Interleukin 1 Alpha	IL1A	1.42	Directly
25	SMAD Family Member 4	SMAD4	19.26	Indirectly
26	KRAS Proto-Oncogene, GTPase	KRAS	19.26	Indirectly
27	Signal Transducer and Activator of Transcription 1	STAT1	19.26	Indirectly
28	TNF Superfamily Member 11	TNFSF11	13.54	Indirectly
29	Inhibitor Of Nuclear Factor Kappa B Kinase Regulatory Subunit Gamma	IKBKG	13.54	Indirectly
30	Major Histocompatibility Complex, Class I, B	HLA-B	13.54	Indirectly
31	Major Histocompatibility Complex, Class II, DR Beta 1	HLA-DRB1	13.54	Indirectly
32	Fibroblast Growth Factor Receptor 1	FGFR1	9.42	Indirectly
33	SOS Ras/Rac Guanine Nucleotide Exchange Factor 1	SOS1	9.42	Indirectly
34	Janus Kinase 2	JAK2	9.32	Indirectly
35	Notch Receptor 1	NOTCH1	7.99	Indirectly
36	Von Hippel-Lindau Tumor Suppressor	VHL	7.99	Indirectly
37	E1A Binding Protein P300	EP300	7.99	Indirectly
38	C-C Motif Chemokine Receptor 6	CCR6	7.92	Indirectly
39	Signal Transducer and Activator of Transcription 3	STAT3	7.92	Indirectly
40	Bone Morphogenetic Protein Receptor Type 2	BMPR2	7.55	Indirectly
41	Interleukin 12B	IL12B	7.06	Indirectly
42	Filamin A	FLNA	6.52	Indirectly
43	Proteinase 3	PRTN3	6.52	Indirectly
44	Transforming Growth Factor Beta Receptor 1	TGFBR1	6.52	Indirectly
45	AKT Serine/Threonine Kinase 1	AKT1	6.08	Indirectly
46	Interleukin 12A	IL12A	5.70	Indirectly
47	Transforming Growth Factor Beta Receptor 2	TGFBR2	5.63	Indirectly
48	Cytochrome P450 Family 1 Subfamily A Member 1	CYP1A1	3.87	Indirectly
49	Catechol-O-Methyltransferase	COMT	3.87	Indirectly
50	B-Raf Proto-Oncogene, Serine/Threonine Kinase	BRAF	5.63	Indirectly
51	WT1 Transcription Factor	WT1	2.85	Indirectly
52	FMRP Translational Regulator 1	FMR1	2.85	Indirectly
53	Solute Carrier Family 2 Member 10	SLC2A10	1.32	Indirectly
54	Collagen Type I Alpha 2 Chain	COL1A2	1.32	Indirectly

### Network of protein–protein interactions

**FIGURE 2:**
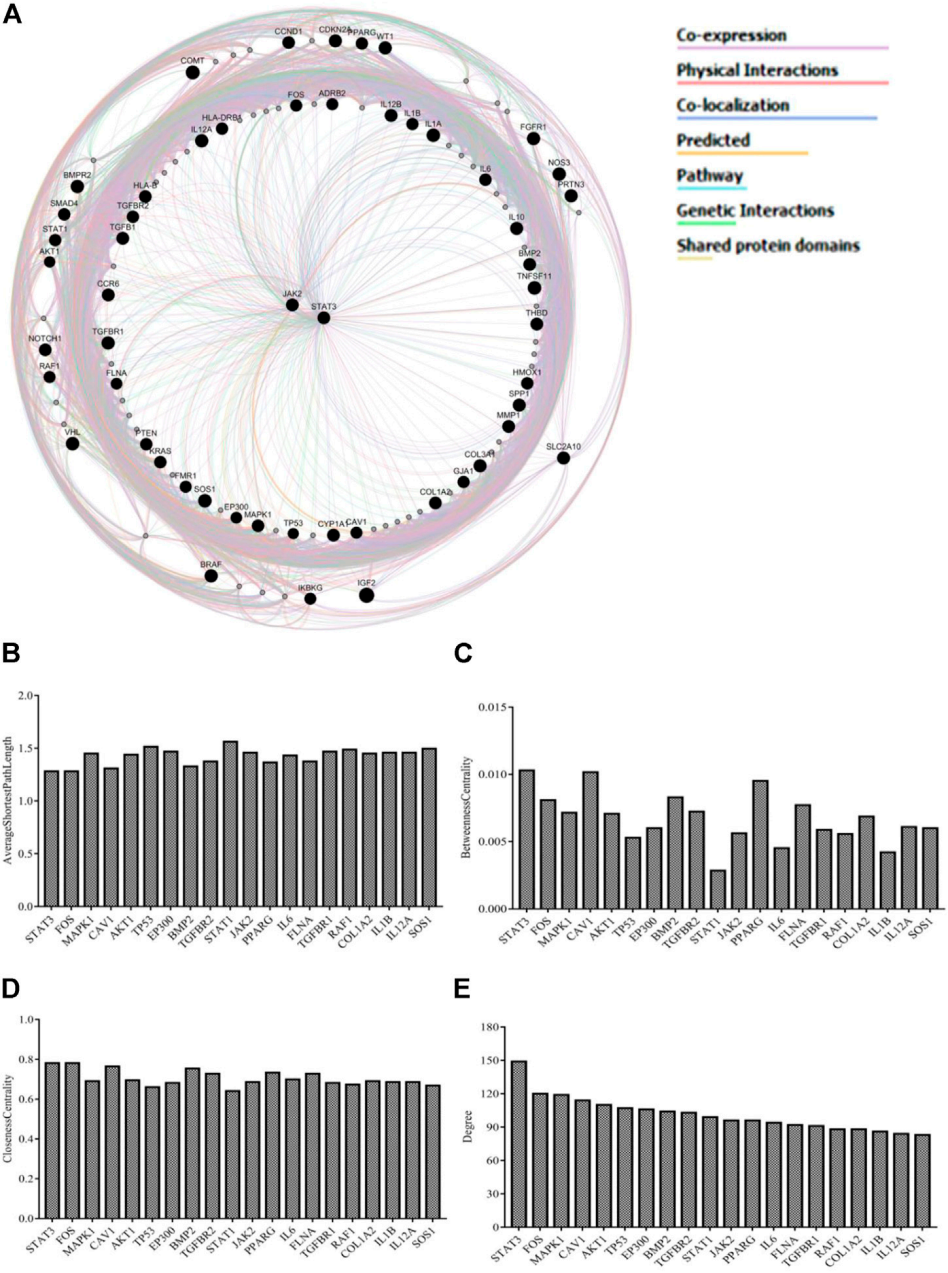
**(A)** GeneMANIA investigated a network of potential quercetin targets against EH. Black-colored genes were used as search terms. Genes associated to query genes are represented by nodes with a lighter black color. Various colors of connecting lines reflect various interrelations, and different functional associations of targets were investigated. The legends in the network shows the types of source networks are grouped together **(B–E)** Topological parameters of the network.

The integrated PPI network was constructed by uploading 54 genes to identify their functional role. The source networks were classified into different categories (e.g., co-expression). The weight of each network along with the number was listed within each category. The weight of interaction correlations in the network is represented by the percentage in the results. According to the outcomes, 55.84% of the target interactions in the network had co-expression and 16.23% had physical interactions. In addition, there were interactions in the form of co-localization (11.27%), predicted (7.37%), pathways (3.92%), genetic interactions (3.31%), and shared protein domains (2.06%) (Figure 2A). Figures 2B–E and Supplementary Table S3 illustrate the estimated average closeness centrality, betweenness centrality, degree, and shortest path length of the nodes in the network. Analysis of the topological variables of the network revealed that genes from the STAT family (STAT1, STAT3) and cytokine-related genes (IL-6, IL-β, and IL-12α) were ranked highly throughout the network.

### Evaluation of gene ontology and pathways

**FIGURE 3:**
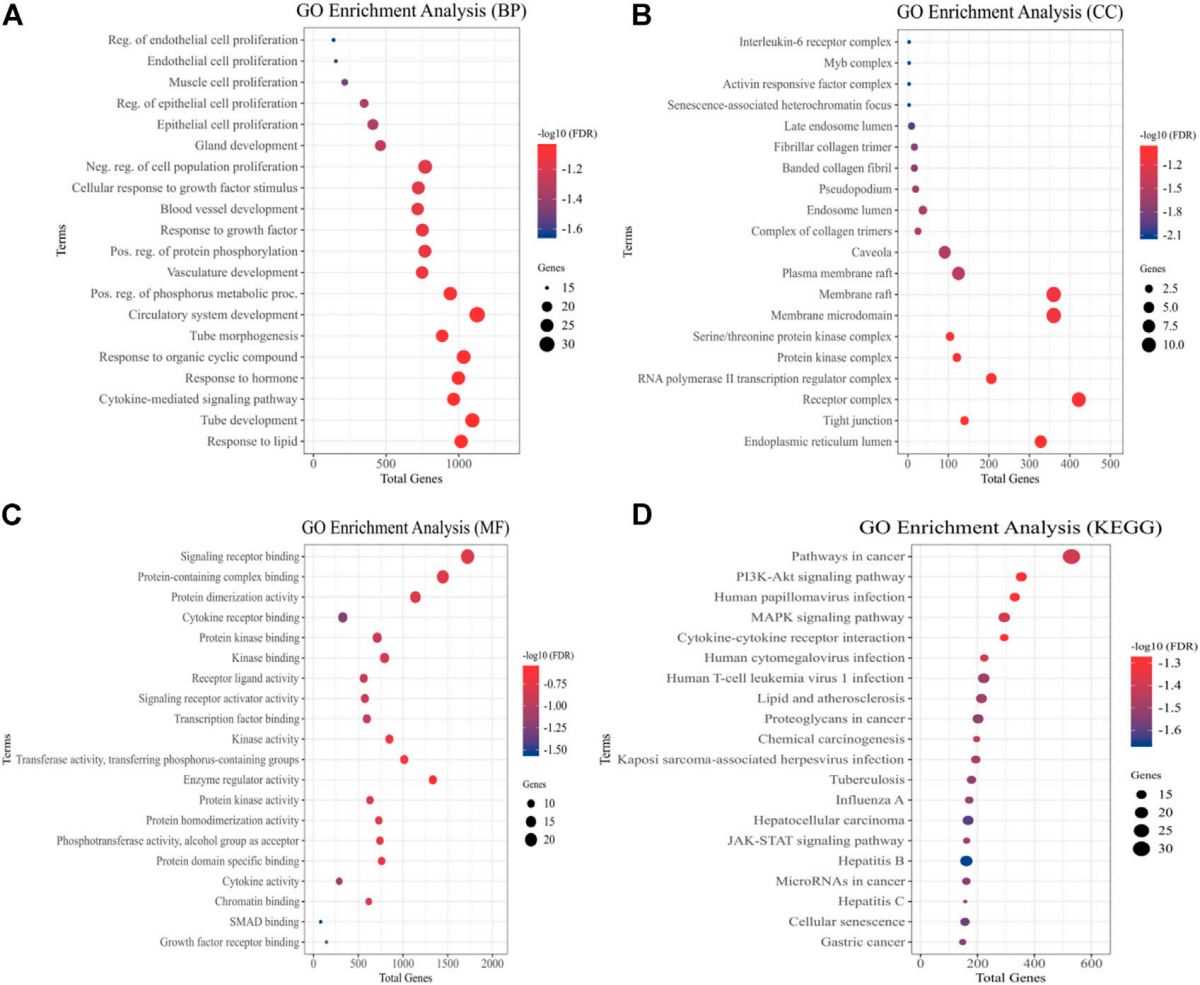
Quercetin targets were analyzed using GO and KEGG pathways. The *y*-axis depicts terms that are greatly enriched, while the enrichment score of these terms is shown on *x*-axis **(A)** Biological Processes (BP), **(B)** Cellular components (CC), **(C)** Molecular Function (MF), and **(D)** KEGG Pathways categories.

We used GO evaluation to further investigate the role of a total of 54 quercetin targets. In the category of EH pathogenesis, these targets were involved in the development of the circulatory system, regulation of endothelial and epithelial cell proliferation, muscle cell proliferation, blood vessel development, and cytokine response (Figure 3A). It also had implication in the plasma membrane raft, RNA Pol-II transcription regulator complex, and IL-6 receptor complex (Figure 3B). In addition, the category of molecular functions included signaling receptor binding, protein kinase binding, cytokines activity, and protein kinase regulator activity (Figure 3C). The effect of quercetin over potential signaling pathways was investigated using KEGG pathway assessment. The analysis showed that “PI3K-AKT signaling pathway,” “cytokine-cytokine receptor interactions,” “JAK-STAT signaling pathway,” “MAPK signaling pathway” and “pathways in cancer” were highly enriched (Figure 3D).

### Networking of target-functions

**FIGURE 4:**
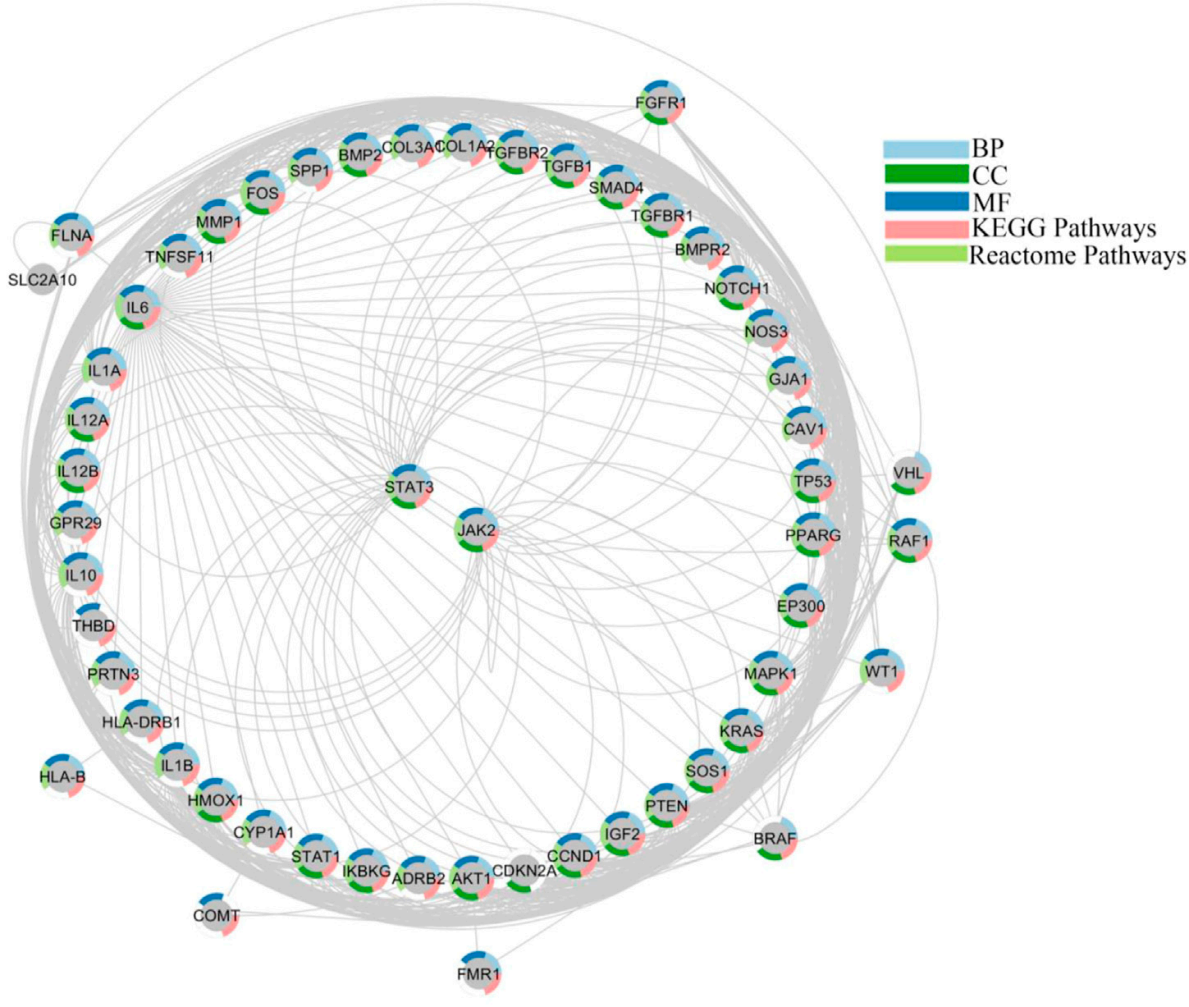
Networking of target-function evaluation. A functional component of interconnected targets is represented in a pie chart with different colors if either the target is actively engaged in GO processes as well as pathways.

We generated the function network/target-pathway after performing extensive network analysis on a number of representative biological processes, molecular function, and signal pathways. Multiple targets were systematically implicated in several biological processes, as given in Figure 4. For instance, STATs, and JAKs were involved in processes like “Wnt signaling pathway,” “JAK/STAT signaling pathway” “protein kinase activity,” “PI3K-AKT signaling pathway,” “vascular smooth muscles contraction,” and “protein homodimerization activity” processes. STAT1 and STAT3 were observed in a number of GO processes, including “circulatory system development,” “cell proliferation regulation,” “cytokine mediated signaling pathway,” “positive regulation of phosphorus metabolic process,” and “cytokine activity”.

### Quercetin inhibits ang II-induced proliferation in vascular smooth muscle cells

**FIGURE 5:**
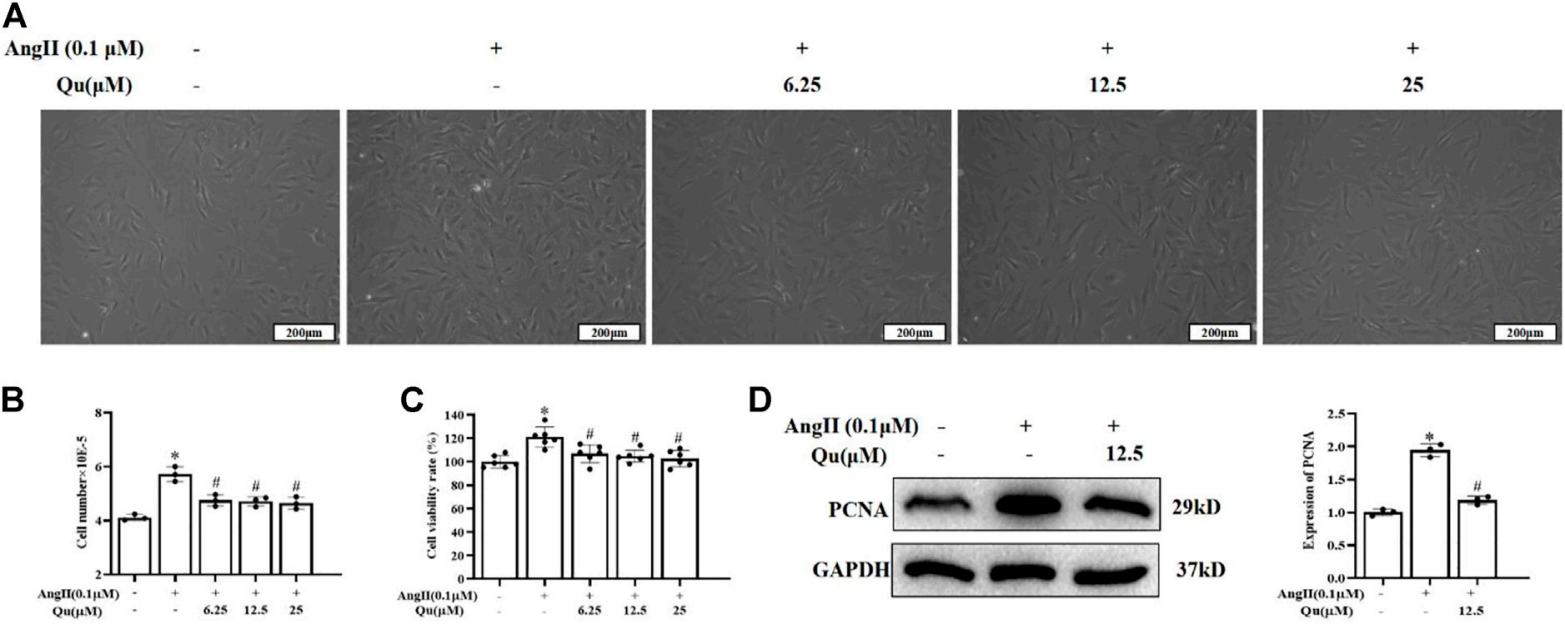
Quercetin’s influences on Ang II-induced VSMCs. 0.1 µM of Ang II stimulated VSMCs were treated with quercetin (6.25, 12.5, and 25 µM) concentrations for 24 h, as shown in **(A)**, descriptive images of the cultured VSMCs. **(B)** Relative number of VSMCs was determined by cell counting. **(C)** VSMCs viabaility was analysed using CCK8 assay. Viability of untreated VSMCs was defined as 100%. **(D)** Indicated PCNA protein expression usingwestern blotting. All values were denoted as mean ± SD; *p < 0.05 Ang II vs. control group, #*p* < 0.05 Ang II + quercetin vs. Ang II group.

The primary VSMCs were isolated from abdominal aorta and identification was performed with α-SMA-antibody and 95% cells were detected positive by immunofluorescence staining (Supplementary Figure S2A). CCK8 assay showed that quercetin treatments (3.125–100 μM) did not affect the cell viability significantly (Supplementary Figure S2B), while Ang II (0.01 and 0.1 μM concentrations) significantly increased cell viability of primary VSMCs (Supplementary Figure S2C). CCK8 assay showed that 6.25, 12.5, and 25 µM concentrations of quercetin treatment attenuated Ang II-induced elevated cell density significantly (Figure 5A), cell number (Figure 5B) and viability of VSMCs (Figure 5C). Ang II stimulation increased the expression levels of PCNA, which was significantly reduced with quercetin treatment (Figure 5D).

### Quercetin inhibited Ang II-induced JAK2/STAT3 activation in vascular smooth muscle cells

**FIGURE 6:**
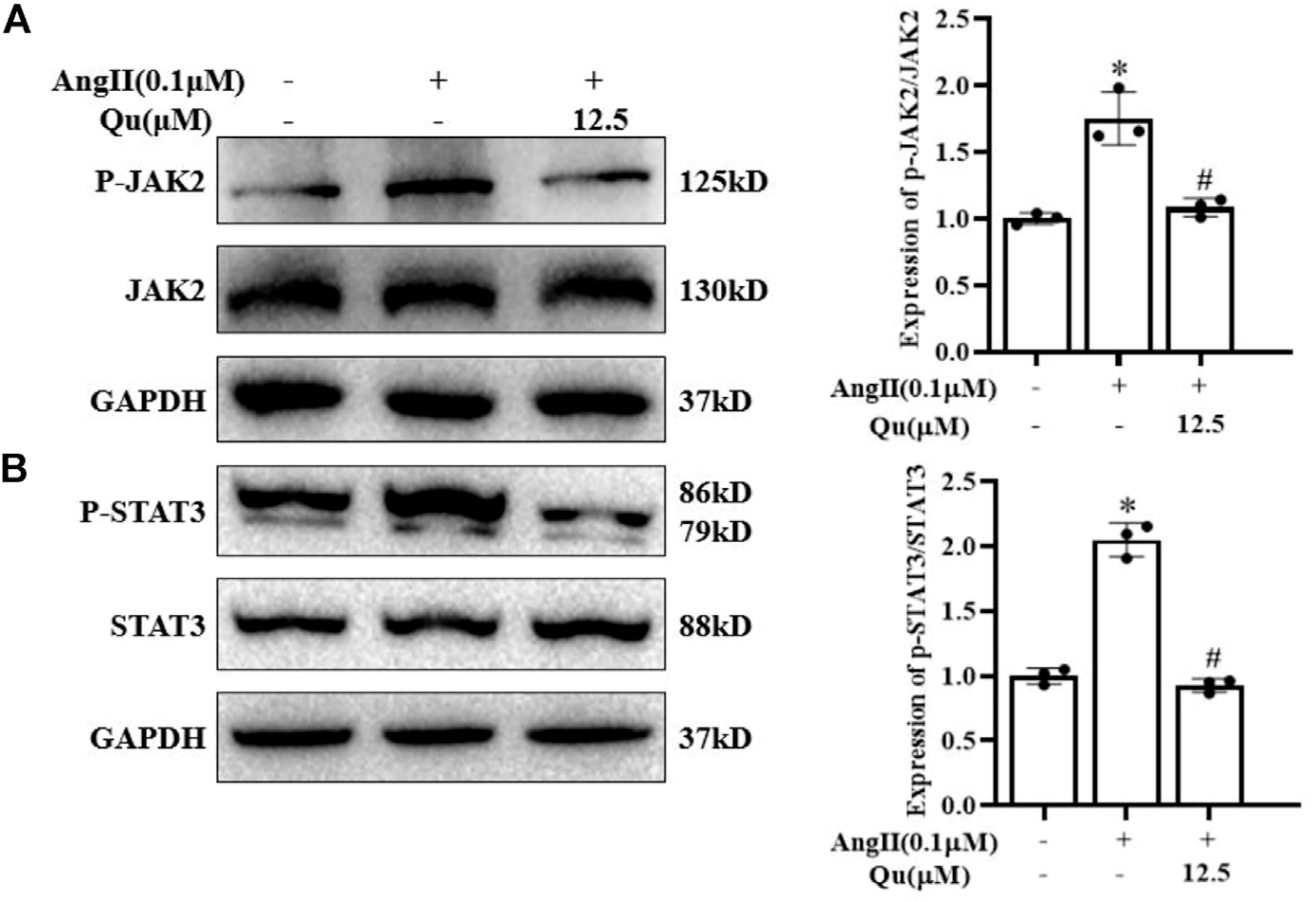
Quercetin attenuates angiotensin-II-induced JAK2/STAT3 activation. **(A–B)** Protein expression of JAK2 and STAT3 along with p-JAK2 and p-STAT3 expression levels was analyzed by western blotting in VSMCs with or without 0.1 µM Ang II-induction and quercetin (12.5 µM) treatment for 24 h **(A)** JAK2 and p-JAK2 protein levels in VSMCs Ang II-induced cells treated with quercetin and quantification of the expression level of p-JAK2/JAK2 (represented in right panel graph) **(B)** STAT3 and p-STAT3 protein levels in VSMCs Ang II-induced cells treated with quercetin and quantification of the expression level of p-STAT3/STAT3 (represented in right panel graph. Data are presented as mean ± SD; *n* = 3 for *in vitro* study; *p < 0.05 vs. Control group, #*p* < 0.05 vs. Ang II group.

Western blot analysis (Figure 6) indicated that stimulation of Ang II activated JAK2 (Figure 6A) and STAT3 (Figure 6B) *via* their phosphorylation in VSMCs, which were attenuated after quercetin (12.5 μM) treatment.

## Discussion

The pathophysiology of EH is a highly complex mechanism. Multiple signaling-associated pathways, including the JAK2/STAT3 pathway, play a significant function in the pathophysiology of EH (Mladěnka et al., 2018; Alanazi and Clark, 2019). Bioavailability of quercetin has been found to be involved in anti-hypertension, specially EH. Therefore, our current study aims to explore the potential underlying mechanism of quercetin on anti-hypertension using network pharmacology analysis. Our results identified 183 quercetin target genes and 384 genes associated with essential hypertension (EH), and 54 common genes of quercetin-EH, which was significantly enriched into several GO processes, i.e., muscle cell proliferation, vascular contraction, regulation of endothelial and epithelial cell proliferation, blood vessel development etc. Furthermore, analysis of the topological variables of the network revealed that genes from the STAT family (STAT1, STAT3), JAK2, and cytokine-related genes were highly ranked throughout the network. KEGG analysis showed the therapeutic effects of quercetin on EH involves PI3K-AKT signaling pathway, JAK-STAT signaling pathway and so on. *In vitro* experiments confirmed that quercetin treatments attenuated the cell density, cell number, and cell viability and reduced PCNA expression in Ang II stimulated VSMCs. Moreover, western blotting analysis showed that quercetin inhibited JAK2/STAT3 activation in Ang II-stimulated VSMCs.

**FIGURE 7:**
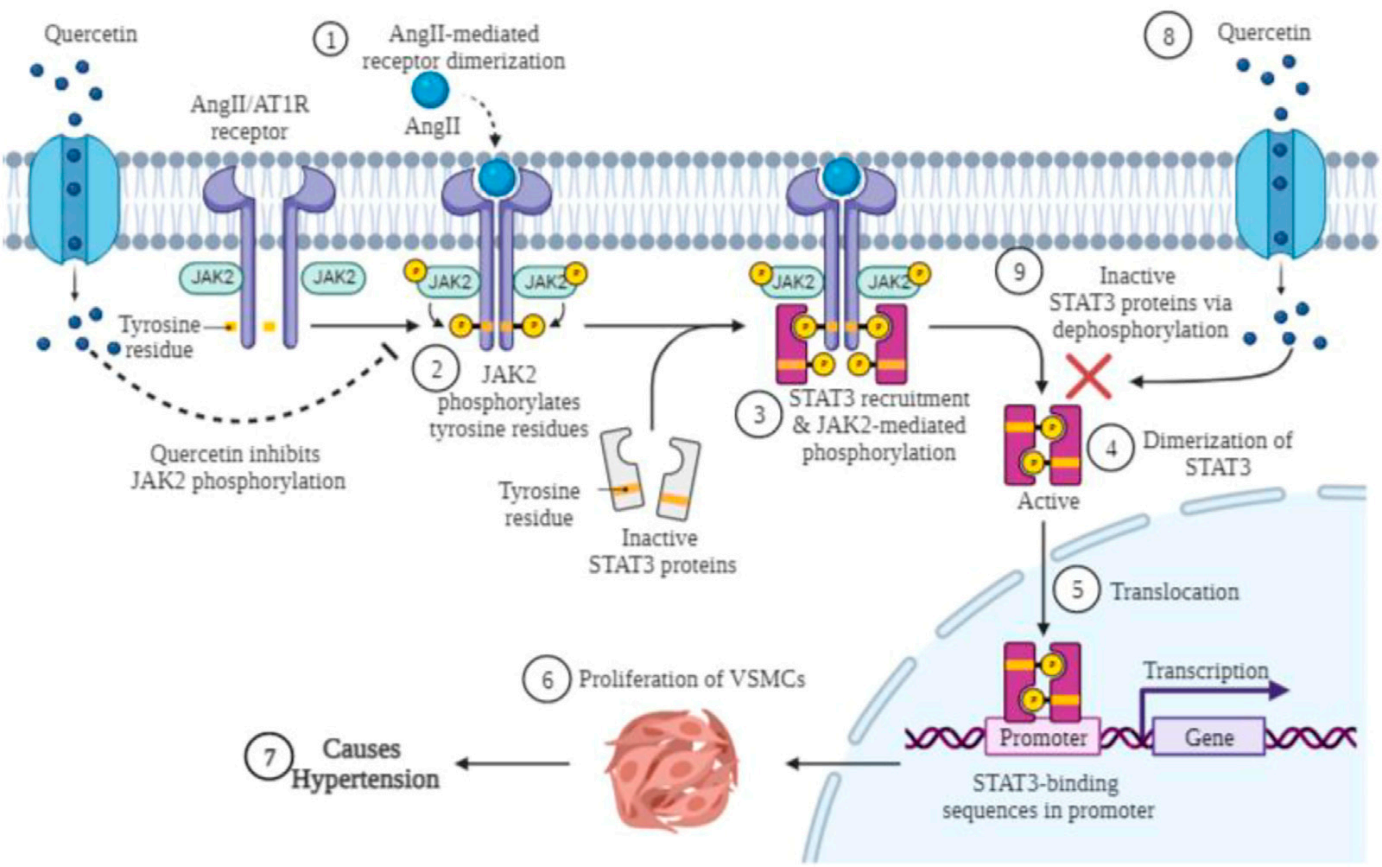
Graphical presentation of anti-proliferative effect of quercetin along with inhibition of JAK/STAT activation in VSMCs. AngII binds to the angiotensin II type 1 receptor (AT1R), which mediates phosphorylation of JAK2, and phosphorylated JAK2 activates the inactivated form of STAT3 by phosphorylation of tyrosine residues. The phosphorylated complex activated by JAK2 and STAT3 assists STAT3 in dimerization. After dimerization of STAT3 protein, it is translocated to the nucleus, where it binds to the gene promoter, and the altered protein expression may lead to proliferation of VSMCs and consequently hypertension. Quercetin treatment inactivates the activated form of JAK2 and STAT3 by dephosphorylation as well as by blocking the dimerization of both proteins. Created with BioRender.com.

Network pharmacology has recently become an interdisciplinary field that encompasses computational biology, conventional pharmacology, structural biology, and multiomics strategies. The overlapping 54 genes of quercetin and EH were entered into the VarElect interactive platform to investigate genotype-phenotype correlations. Analysis of the topological variables of the network revealed that genes from the STAT family, cytokine-related and nitric oxide associated genes were highly ranked throughout the network. The family of NOS enzymes are categorized into three NOS subgroups: NOS1, NOS2, and NOS3. All such subgroups are participating at a large scale in physiological processes within the central nervous system, immune, as well as cardiovascular system (Simons et al., 2016). The PPARY has been linked to the pathogenicity of a variety of diseases namely obesity, diabetes, high blood pressure, as well as tumors; it furthermore plays an essential part in the pathology of EH (Echeverría et al., 2016). JAK2 and STAT3 genes were also observed with VarElect implemented scores (9.32 and 7.92 respectively) among the key genes. Moreover, quercetin and EH overlapping targets revealed that the highly enriched signaling pathways were attributed to the regulation of endo/epithelial cell proliferation and signaling pathways. STATs and JAKs were involved in several GO and signaling pathways, such as cancer pathways, JAK/STAT signaling pathway, AKT signaling pathway, vascular smooth muscle contraction, and so on. STAT1, STAT3, and JAK2 have been observed in various GO processes, including development of the circulatory system, regulation of cell proliferation, cytokine-mediated signaling, and cytokine activity. All of these signaling pathways are involved in the development of EH. The GO enrichment and KEGG pathways analyses have been uniformed with the VarElect analysis’s correlation between targets as well as phenotype. Based on the findings of the aforementioned studies, it is clear that quercetin has an important therapeutic and preventive role in EH caused by multiple targets (Jakaria et al., 2019). The JAK/STAT pathway responds to growth factors and cytokines by transducing signals from the cell surface to the nucleus in various cell types, such as smooth muscle proliferation, endothelial dysfunction, and inflammation (Lacolley et al., 2018; Liu et al., 2019). JAK is a receptor for several members of the STAT family, which includes STAT3. JAK2 and its associated receptors and STAT3 pathway are activated by Ang II (He et al., 2019). JAK2 activation has been associated with VSMCs proliferation, vascular endothelial apoptosis, and vascular cell contraction *in vitro* and *ex vivo* experiments (Zhang et al., 2018). While Ang II bind to their specific cell surface receptors and then recruit and activate receptor-associated JAKs (Gai et al., 2021). The activated JAKs phosphorylate tyrosine amino acids from specific receptors (like, AT1R) that identify SH2 domains of STATs (Bousoik and Montazeri Aliabadi, 2018). Once phosphorylated, STAT3 help to form the STAT homo/hetero dimers and transport them to the nucleus, where they adhere with specific DNA sequences and alter the expression of the genes involved (Bhaskaran et al., 2014; Harhous et al., 2019). The mechanistic representation is shown in Figure 7. In previous studies, inhibiting this route reduced neointima development and cell proliferation after intima disruption in the specific carotid arteries (Milara et al., 2018). The importance of STAT3 in vascular dysfunction disorders have been hampered due to insufficient progress of genomic studies or specific pharmacological antagonists. This suggests that the JAK/STAT signaling pathway may play a significant role in the control of vascular function through Ang II (Jamilloux et al., 2019).

Furthermore, for research community, flavonoids are the keen interests because of their diverse nature in pharmacological and biological reasons; such as anti-inflammatory, antioxidant, antiviral, and anticancer activities (Agrawal, 2011; David et al., 2016). In addition to above properties, quercetin is found in derivative forms in human plasma including glucuronide as well as α-tocopherol and α/β-carotene. The significantly higher levels of α-tocopherol and about 1% quercetin were found in isolated low-density lipoprotein (LDL) from human plasma after onion consumption (Moon et al., 2000). Based on Cu^2+^-induced oxidation of human LDL, quercetin derivatives including 3P-O-methyl quercetin showed a prolonged lag phase with a half scale of effect compared to that of aglycone (Manach et al., 1998). Additionally, two quercetin conjugates were studied in the plasma of quercetin-administrated rats, namely quercetin 3-O-β-D-glucuronide (Q3GA) and quercetin 4′-O-β-D-glucuronide (Q4′GA). Orally administered Q3GA had an antioxidant effect in isolated LDL from rat plasma as well as on Cu^2+^-induced oxidation of LDL in human plasma (Moon et al., 2001). Quercetin has been studied widely in a variety of animals for the treatment of several disorders *via* targeting multiple signaling pathways. Ang II induced activation of MAP kinase ERK1/2, JNK, and p38 in rat aortic smooth muscle (RASMC). In RASMC induced by Ang II, quercetin has an inhibitory effect on JNK, whereas ERK1/2 and p38 activation were not affected by quercetin treatment (Yoshizumi et al., 2001; Yoshizumi et al., 2002; Perez-Vizcaino et al., 2006). In several *in vivo* animal models, it was investigated that dimerization and translocation of p-STAT3 occur through phosphorylation of the amino acid Tyr-705 catalyzed by JAK1, JAK2, JAK3, and TYK2, whereas Ser-727 is phosphorylated by MAPK, which maximizes the transcriptional capacity of STAT3. Moreover, in 1-methyl-4-phenyl-1,2,3,6-tetrahydropyridine (MPTP) models, STAT3 activation was found to be associated with JAK2 in astrocytes, whereas MPTP activated ERK1/2 phosphorylation, did not lead to phosphorylation of STAT3 at the Ser-727 residue (Skaper et al., 2001; Yanagisawa et al., 2001; Sriram et al., 2004).

Moreover, the influences of Ang II on phosphorylation of STAT3 in primary cultured brain stem cells were identical to the effects of the peptides (Kandalam and Clark, 2010). The stimulation of JAK2/STAT3 pathway *via* Ang II has been shown in experimental studies, both *in vitro* and *in vivo*, and therefore play a vital role in the development of Ang II-dependent hypertension (Satou and Gonzalez-Villalobos, 2012). In VSMCs, Ang II induces JAK2, which activates RhoA guanine nucleotide exchange factor I, Arhgef1, which eventually activates RhoA signaling and causes hypertension (Banes-Berceli et al., 2011). In contrast, suppression of Arhgef1 reduces Ang II-induced hypertension (Terada and Yayama, 2021). Moreover, therapeutic suppression of JAK2 has been shown to reduce the progression of hypertension in Ang II-infused rats (Guilluy et al., 2010; Kirabo et al., 2011). The same effect was observed in JAK2 knockdown cells (Kirabo et al., 2011). *In vivo* results also shown that modulation of JAK2 and AT1Rs reduces the progression of hypertension and proteinuria in diabetic renal dysfunction (Forrester et al., 2018; El-Arif et al., 2022). In myocytes, Ang II triggered biphasic STAT3 phosphorylation regulated by TLR4 (Han et al., 2018). Our current study revealed that quercetin inhibits the proliferation and JAK2/STAT3 pathway activation in Ang II stimulated VSMCs. Comparative to the previous finding in terms of association of Ang II with JAK/STAT signaling, our results suggest that quercetin inhibits the proliferation and plays a significant role in inhibiting the JAK2/STAT3 activation via their phosphorylation in VSMCs induced by Ang II.

## Conclusion

In this current study, networking pharmacology approach revealed that quercetin exerts its effect through multiple signaling pathways against EH targets. Moreover, *in vitro* studies showed that quercetin inhibits the proliferation of VSMCs targeting JAK2/STAT3 signaling induced by Ang II.

## Data Availability

The datasets presented in this study can be found in online repositories. The names of the repository/repositories and accession number(s) can be found in the article/Supplementary Material.
