# Supplementary material for: Quercetin inhibits angiotensin II-induced vascular smooth muscle cell proliferation and activation of JAK2/STAT3 pathway: A target based networking pharmacology approach
Source: Front Pharmacol. 2022 Oct 17;13:1002363. doi: 10.3389/fphar.2022.1002363 (PMC9618806; doi:10.3389/fphar.2022.1002363)
Supplement: Supplementary file 1 [file DataSheet2.ZIP › Supplemetary files/Suppementary data.docx]

**
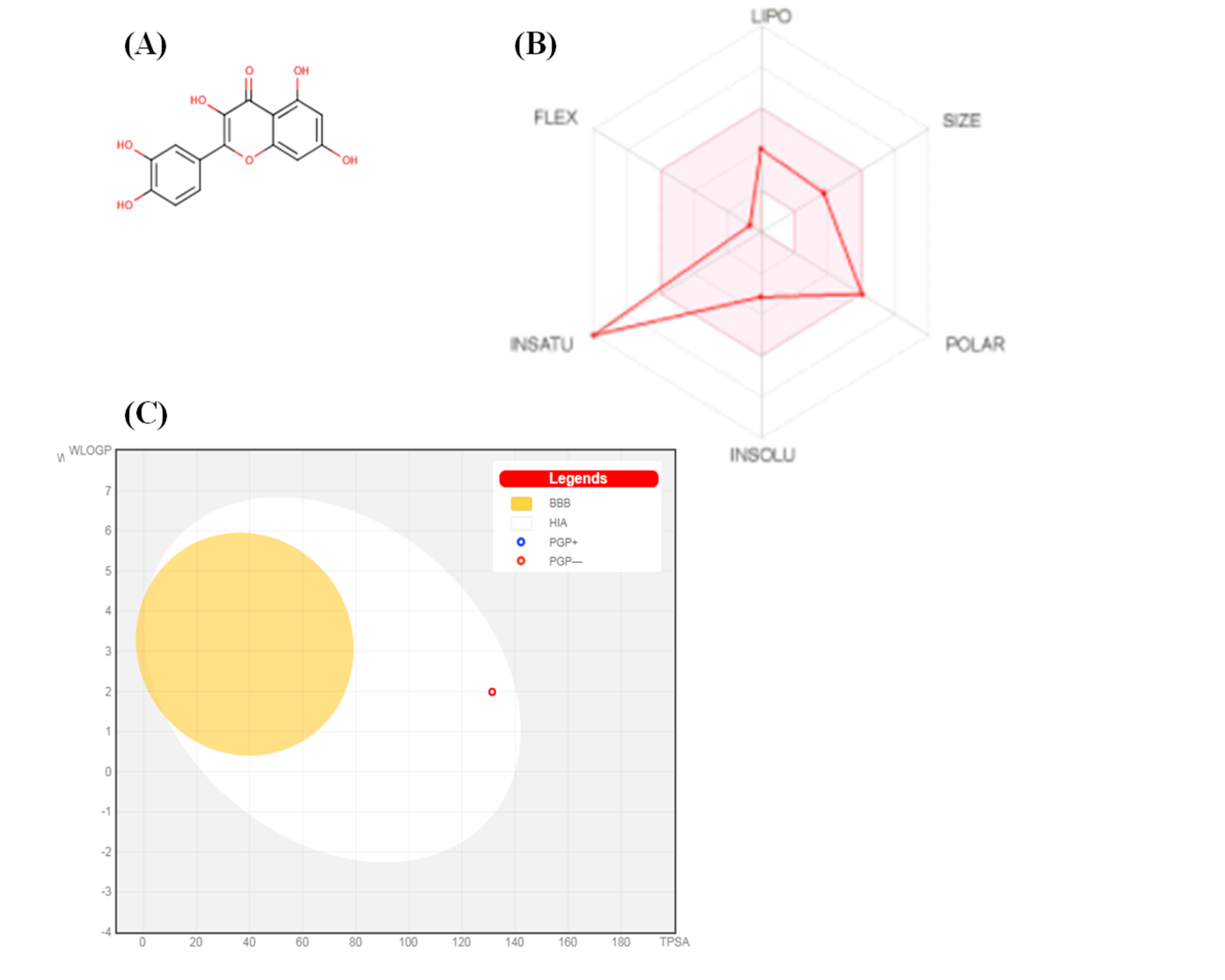
**

**FigureS1.** Analysis of pharmacokinetics of Quercetin **(A)** chemical structure **(B)** physicochemical properties **(C)** Quercetin's gastrointestinal absorption and brain penetration anticipated by a BOILED-EGG analysis.


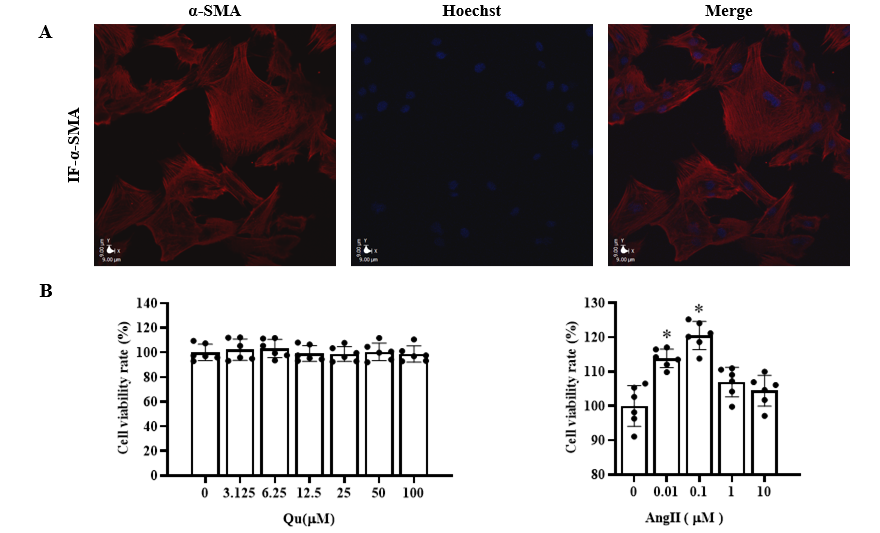


**Figure. S2.** (A) The effects of Quercetin on AngII-stimulated vascular smooth muscle cells (VSMCs). Immunofluorescent (IF) staining was used to determine survivability of extracted VSMCs by using α-SMA and Hochest staining. (B-C) CCK8 assay was performed to determine the cell viability of VSMCs after treatment with Quercetin (3.125-100μM) or 0.01-10µM AngII used to stimulate VSMCs for 24 hours. *p<0.05AngII vs. control (zero) group.

**Table S1.** Summary of targets


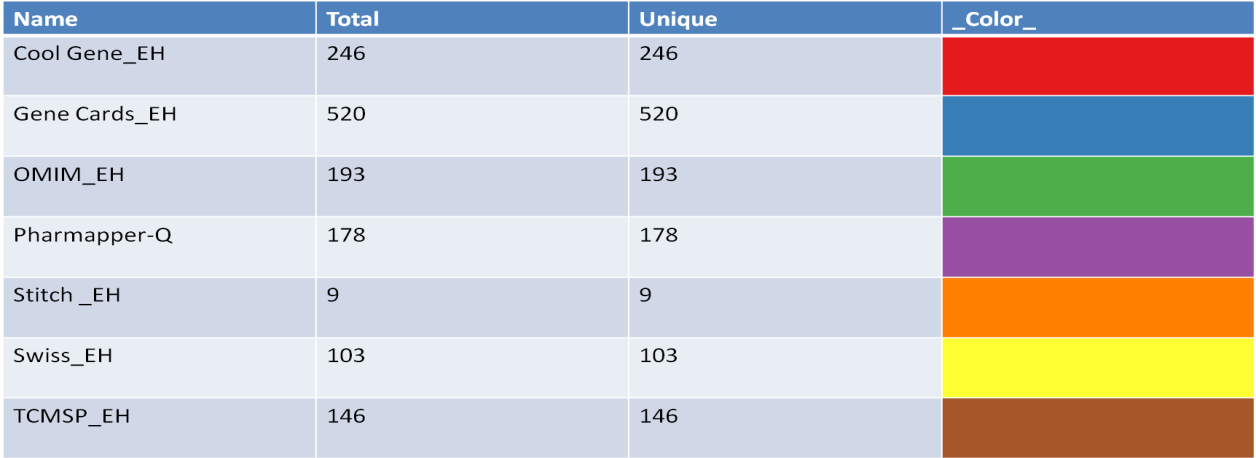


**Table S2**. Targets retrieved from various databases and redundant targets were removed. Final list of genes/ targets are shown in UniProt IDs.

| **Pharmapper-Quercetin** | **TCMSP_EH** | **Swiss_EH** | **OMIM_EH** | **Gene Cards_EH** | **Cool Gene_EH** | **Stitch _EH** |
| --- | --- | --- | --- | --- | --- | --- |
| P06276 | Q9UNQ0 | Q9NPH5 | P05161 | Q13873 | P05305 | P08631 |
| P26196 | Q13085 | P30518 | O00468 | P12821 | Q13873 | P10632 |
| P00918 | P22303 | P15121 | P43489 | P37023 | O76074 | P04798 |
| P04062 | P07550 | P47989 | Q96L58 | P17813 | P29474 | Q16678 |
| O15382 | P35869 | P21397 | P51172 | P11686 | P15692 | P11168 |
| P11309 | P15121 | P08069 | O14640 | O14649 | P16860 | O94768 |
| P24941 | P31749 | P36888 | Q96S94 | P01019 | P01019 | Q6NVY1 |
| P45452 | P09917 | P11511 | Q5T2N8 | P29474 | P00797 | Q07820 |
| P00734 | P10275 | P00533 | Q5T9A4 | O14746 | P05231 | P11309 |
| P03950 | P10415 | P00734 | Q9NVI7 | P30556 | Q03135 |  |
| Q08499 | Q07817 | P00918 | Q96AX9 | P07988 | P35318 |  |
| Q07343 | O15392 | P11309 | O95544 | Q03135 | P02741 |  |
| P00491 | P12643 | P09917 | Q05513 | P05305 | P37023 |  |
| P07858 | P42574 | Q96GD4 | P12755 | Q99758 | Q16665 |  |
| P05451 | Q14790 | P21917 | O75038 | Q9P2K8 | P02768 |  |
| P33176 | P04040 | P30542 | Q92956 | P00797 | P12821 |  |
| Q16539 | Q03135 | P43166 | Q9HAZ2 | O15198 | P01137 |  |
| P43235 | P13500 | Q04760 | O75095 | P01375 | P61586 |  |
| P42330 | P24385 | P05164 | Q9P2S5 | P57082 | P31749 |  |
| P15121 | P29965 | P27986 | O15350 | P05231 | Q9BYF1 |  |
| P19367 | P38936 | P29274 | B2RUZ4 | Q9NZ71 | Q9ULZ1 |  |
| P23368 | P42771 | P53355 | Q8N1G4 | P01137 | P35228 |  |
| P35221 | Q8N726 | P06737 | O75161 | Q8IWL2 | P25101 |  |
| P00439 | O96017 | P00915 | Q93038 | Q12946 | P01282 |  |
| P50579 | O15111 | P49841 | O94827 | P16860 | Q06889 |  |
| Q04609 | P02452 | P12931 | Q99497 | P01160 | P09601 |  |
| P11172 | P02461 | Q05397 | Q9UJM3 | Q8IWL1 | P22001 |  |
| P35558 | P02741 | P37059 | Q9Y2W3 | P02768 | P01375 |  |
| Q00796 | P07339 | P35968 | Q9BSK2 | P37231 | P17813 |  |
| P20248 | P02778 | P45452 | O94985 | D6RA96 | P41180 |  |
| P60174 | P10145 | P08254 | Q9NSA3 | P02741 | P31645 |  |
| P15309 | P04798 | P07451 | O60333 | P16520 | P22460 |  |
| P12724 | P05177 | P16050 | Q86V15 | P54278 | P15502 |  |
| P09211 | Q16678 | P33527 | Q13148 | P19099 | P17931 |  |
| P02768 | P08684 | P53350 | O00187 | P10747 | P01258 |  |
| P00742 | Q9NRD8 | P23280 | P42345 | P35247 | P06881 |  |
| P04075 | P01133 | P06493 | Q9P2K9 | Q9NP81 | P13500 |  |
| P60568 | P00533 | P14780 | P42898 | P03956 | Q9Y210 |  |
| Q08881 | P56537 | O43570 | P01160 | P35611 | P28482 |  |
| P23526 | P04626 | P08253 | P16860 | P05121 | P01588 |  |
| P34896 | P21860 | Q16512 | Q02809 | P42898 | O14649 |  |
| P08254 | P03372 | Q9ULX7 | P20333 | P09601 | P08519 |  |
| Q9UM07 | Q92731 | Q16790 | O75911 | P01009 | P24530 |  |
| P42574 | P13726 | P68400 | Q86YL7 | P01308 | P09429 |  |
| P12931 | P01100 | P18054 | Q96C19 | P15692 | P37231 |  |
| Q10588 | P17302 | P08581 | P08217 | Q9Y2X3 | P42345 |  |
| P18031 | P49841 | P22748 | Q8WUP2 | P00734 | O95399 |  |
| P22894 | P09488 | P51955 | Q96T58 | P07550 | Q16647 |  |
| Q02127 | P28161 | P25024 | Q9UBY9 | O95453 | P01009 |  |
| P00533 | P09211 | Q13554 | P51801 | P15502 | Q15389 |  |
| P08246 | Q16665 | Q9UM73 | P29317 | P14780 | Q9NPH5 |  |
| P00749 | P09601 | P31749 | Q6P3S6 | P22301 | Q99988 |  |
| P05019 | Q00613 | P08183 | P55001 | Q9HC84 | P14174 |  |
| P15086 | P11021 | Q9HC98 | Q9NQ11 | P08235 | P35354 |  |
| O15530 | P04792 | P04054 | P21912 | P12259 | Q9UK05 |  |
| P62942 | P05362 | P35218 | Q9ULC6 | Q5T7M9 | P08473 |  |
| P06744 | P01579 | P56817 | Q6TGC4 | P10145 | P48061 |  |
| Q16775 | P01344 | Q16678 | O95154 | Q16647 | P14210 |  |
| P0CG30 | P17936 | P30530 | P41271 | Q53TS8 | P40763 |  |
| Q9UKM7 | P22301 | Q9UNQ0 | Q9NZK7 | P13500 | P01185 |  |
| P17752 | P01583 | O60285 | P14555 | P01584 | Q15848 |  |
| P49137 | P01584 | P52895 | P39877 | P43699 | P07996 |  |
| P11712 | P60568 | Q04828 | Q9BZM2 | P07204 | Q9UM47 |  |
| P20132 | P05231 | P42330 | Q969V5 | P25101 | O00206 |  |
| P05162 | P06213 | P17516 | P42892 | P01579 | Q9HBA0 |  |
| P35968 | P10914 | Q8N1Q1 | P05186 | P35228 | P35414 |  |
| O60674 | P05412 | P14550 | Q86UV5 | P01911 | Q99814 |  |
| Q93088 | Q12809 | Q9HC97 | P98160 | P21359 | P00441 |  |
| O00204 | P27338 | P43405 | P60953 | P98161 | P41159 |  |
| P55263 | P28482 | P10636 | P56705 | O94988 | P60484 |  |
| P07359 | P27361 | B2RXH2 | P29322 | O00206 | P09038 |  |
| Q08188 | O43451 | P11388 | P02745 | Q13485 | P09619 |  |
| P00374 | P03956 | P06213 | P02747 | P41159 | P07204 |  |
| O60760 | P08253 | P22303 | P02746 | P04275 | Q13976 |  |
| P49888 | P08254 | Q15746 | P29323 | P31327 | P78423 |  |
| P14780 | P14780 | P48736 | Q8TDY4 | Q15848 | P39210 |  |
| O76074 | P05164 | P27695 | Q02535 | P35318 | Q02763 |  |
| P00492 | P01106 | Q15078 | Q8NDY4 | P42892 | P69892 |  |
| P13501 | P14598 | Q00535 | P62913 | P00750 | P01042 |  |
| Q9BZX2 | P25963 | Q8WWL7 | P34972 | P02649 | P04035 |  |
| P07900 | P35228 | P14635 | Q8IU57 | P29279 | Q9UBU3 |  |
| P04181 | P29474 | O95067 | Q5TH74 | P17948 | P38936 |  |
| P49773 | P55786 | P05089 | Q9UKA8 | Q14432 | P12644 |  |
| P11766 | P15559 | Q13332 | Q9Y696 | Q16665 | P08246 |  |
| P08473 | O75469 | Q92731 | Q13761 | P31645 | P08253 |  |
| P39900 | Q14994 | P29372 | Q8N5G2 | P00533 | P49763 |  |
| P06737 | P11926 | Q96S37 | Q5SW96 | P00441 | P14780 |  |
| P08263 | P09874 | Q00534 | Q9NR34 | P01042 | P00533 |  |
| P23946 | P00750 | P24941 | Q9NZV5 | P22004 | P05164 |  |
| P78417 | P00749 | P14679 | Q9BRI3 | P31749 | Q9HD89 |  |
| P29317 | P27169 | P14061 | Q7RTX7 | P80365 | P10451 |  |
| P49759 | P16435 | P35869 | Q5T124 | P05362 | Q9Y2G2 |  |
| P32589 | Q07869 | P11474 | Q15466 | P08246 | P35968 |  |
| P04035 | Q03181 | P05067 | P19634 | P01008 | Q9P2K8 |  |
| P00797 | P37231 | P09874 | O75636 | P15509 | Q15109 |  |
| O14727 | P17252 | P02766 | P46089 | P43694 | Q15796 |  |
| P56817 | P05771 | P39900 | Q9Y6W5 | P16109 | P05362 |  |
| P14324 | P07477 | P28907 | Q86Y82 | O76074 | A0A1W2PQU2 | |
| P10276 | P60484 | O60218 | P25105 | P20815 | P84022 |  |
| Q92947 | P43115 | Q9H2K2 | Q9UII2 | P01889 | P07900 |  |
| P00517 | P23219 | O95271 | Q9BV79 | P01033 | P11413 |  |
| Q8IV48 | P35354 | P11387 | Q13571 | P08253 | P00750 |  |
| Q9NPB1 | P04049 | O14746 | O75056 | O00255 | P27361 |  |
| P23919 | P20936 |  | Q96SA4 | P15538 | Q16539 |  |
| P31939 | Q9NS23 |  | Q9GZM7 | P13569 | P10721 |  |
| P20231 | P06400 |  | Q07666 | P13726 | O15392 |  |
| P61587 | Q04206 |  | Q9BUN5 | Q9H668 | P61073 |  |
| P23381 | Q13950 |  | Q13547 | Q13563 | P57082 |  |
| P53779 | P19793 |  | Q9H7C4 | Q92908 | P26447 |  |
| P30613 | Q14524 |  | P54577 | P04637 | P04179 |  |
| P23677 | P16581 |  | Q96BU1 | P05164 | P29475 |  |
| P18085 | P05121 |  | Q8NAU1 | P24530 | P48995 |  |
| P12268 | P00441 |  | P54819 | P35354 | P62937 |  |
| P28161 | P42224 |  | Q96A70 | Q96J92 | Q9NYA1 |  |
| P49841 | P49888 |  | Q7Z408 | P51168 | P04637 |  |
| P16581 | P01137 |  | Q8WW32 | P04150 | O60674 |  |
| P06239 | P07204 |  | P35212 | P15924 | Q5XXA6 |  |
| Q16772 | P01375 |  | Q9UIF3 | P35555 | P17948 |  |
| Q96T66 | P11387 |  | Q5D1E8 | O60674 | P13726 |  |
| Q9Y689 | P11388 |  | Q8TAD8 | P84022 | P26022 |  |
| P60953 | P04637 |  | Q2MKA7 | P01258 | Q07954 |  |
| P16442 | P19320 |  | Q9NX04 | P06881 | P41595 |  |
| Q9H4M7 | P15692 |  | Q86U90 | Q9H4A3 | P17752 |  |
| P09488 | P47989 |  | Q12874 | P50052 | O60565 |  |
| P35520 | P14635 |  | Q13643 | P09488 | O00300 |  |
| P52848 | P19419 |  | Q99417 | P02751 | O94760 |  |
| Q13838 | Q07812 |  | Q9UPN3 | Q8N2S1 | P21359 |  |
| P09960 | P49895 |  | Q9UNP9 | P52952 | O15198 |  |
| P36959 | Q01094 |  | Q9H3H1 | P13501 | P35225 |  |
| P51151 | Q14209 |  | P12524 | P05112 | P05019 |  |
| P00390 | O14493 |  | Q8NA29 | P05019 | P46937 |  |
| O14965 | P55211 |  | P50897 | P46531 | Q01638 |  |
| P10153 | Q06455 |  | Q13129 | Q9Y5Q5 | P34932 |  |
| P07686 | Q92819 |  | O75844 | P01583 | P20366 |  |
| P42224 | P52789 |  | Q96GD3 | Q9BYF1 | P01160 |  |
| P29466 | P19875 |  | Q9HAB8 | P35225 | P12643 |  |
| Q7Z4W1 | Q16236 |  | Q32P28 | P37088 | P10145 |  |
| P03951 | Q99801 |  | Q96PL5 | P19320 | P22301 |  |
| O43708 | O14625 |  | Q96MR6 | P51170 | Q12968 |  |
| Q16836 | Q15113 |  | Q9BW60 | P09038 | P04070 |  |
| P50613 | O43242 |  | Q5T011 | P36897 | P19838 |  |
| Q92831 | P14672 |  | P10586 | Q14116 | Q92736 |  |
| P06730 | P08294 |  | Q11203 | P10451 | Q9GZT9 |  |
| P22392 | P10451 |  | P48067 | P10645 | P10415 |  |
| P68106 | O95433 |  | Q99661 | P08254 | O00429 |  |
| P00480 | Q96JK2 |  | Q8NFU0 | P00748 | Q14721 |  |
| P20339 | Q9UII4 |  | Q9H4B4 | P16581 | P39060 |  |
| Q02750 |  |  | Q9Y6C5 | P04141 | P01574 |  |
| Q03518 |  |  | Q9UIF7 | P08294 | P34913 |  |
| Q06520 |  |  | Q9Y4U1 | P04062 | Q96P20 |  |
| P61626 |  |  | Q06830 | P35222 | P01584 |  |
| Q06187 |  |  | Q6P0Q8 | P22460 | P07492 |  |
| P10275 |  |  | Q92698 | P01116 | Q08050 |  |
| P52732 |  |  | Q15345 | P29400 | P16284 |  |
| O14717 |  |  | P07919 | Q15109 | P19429 |  |
| Q9P2T1 |  |  | O00519 | P48544 | Q96EB6 |  |
| P53355 |  |  | A6PVL3 | P18510 | Q12778 |  |
| P02679 |  |  | P13584 | P98196 | P32418 |  |
| Q9D4P0 |  |  | Q02928 | Q86TI2 | Q12946 |  |
| P31749 |  |  | Q13113 | Q9UK05 | P43694 |  |
| Q15075 |  |  | Q13461 | P35968 | P23582 |  |
| P33316 |  |  | P26378 | P02647 | P78348 |  |
| Q16222 |  |  | Q96SU4 | Q6ZTR7 | P02649 |  |
| P62826 |  |  | Q13415 | P01588 | P21399 |  |
| P00568 |  |  | Q5TAX3 | O60603 | P05112 |  |
| P35228 |  |  | P23786 | P12643 | P03956 |  |
| P07953 |  |  | Q8NBF1 | P17302 | O75116 |  |
| Q969G6 |  |  | Q8WXI4 | P49763 | P42574 |  |
| Q96C86 |  |  | O95801 | P06858 | P21980 |  |
| P50225 |  |  | Q15392 | Q9NPH5 | P30556 |  |
| P11086 |  |  | Q8NBP7 | P15941 | P17342 |  |
| P63000 |  |  | Q9UPU5 | P37173 | P16220 |  |
| P08709 |  |  | P09758 | P40763 | Q14116 |  |
| Q08257 |  |  | Q5VVJ2 | O15539 | P80188 |  |
| P10114 |  |  | Q96C11 | P02671 | Q9BQ08 |  |
| P01112 |  |  | Q12857 | P04040 | P14138 |  |
| P35270 |  |  | Q5T7N3 | P61278 | P12931 |  |
| Q9NXA8 |  |  | Q9Y5C1 | P78504 | P12259 |  |
| Q9BZ11 |  |  | Q9Y672 | Q15389 | P47985 |  |
| Q00688 |  |  | Q01973 | Q9HD89 | P46531 |  |
| P06213 |  |  | Q9HCJ3 | P62736 | P11309 |  |
| P14061 |  |  | P27144 | Q16558 | P01135 |  |
|  |  |  | P48357 | Q8N2X6 | P36897 |  |
|  |  |  | O15243 | P61586 | P49961 |  |
|  |  |  | Q5VTH9 | P02452 | P18146 |  |
|  |  |  | Q99665 | Q06124 | O15553 |  |
|  |  |  | P24522 | P28223 | P42892 |  |
|  |  |  | O95661 | P00738 | Q08722 |  |
|  |  |  | P32929 | O95399 | P04040 |  |
|  |  |  | P43115 | P19429 | P36894 |  |
|  |  |  | O95218 | P47989 | Q15672 |  |
|  |  |  | Q9BVH7 | P10147 | P49908 |  |
|  |  |  | Q8TEY7 | P04114 | P08294 |  |
|  |  |  |  | Q16552 | P23946 |  |
|  |  |  |  | P13498 | P07225 |  |
|  |  |  |  | P08588 | O95865 |  |
|  |  |  |  | P68032 | P01033 |  |
|  |  |  |  | P11473 | P01562 |  |
|  |  |  |  | P01282 | P22736 |  |
|  |  |  |  | Q99593 | P24821 |  |
|  |  |  |  | P05026 | P18075 |  |
|  |  |  |  | P27815 | P29274 |  |
|  |  |  |  | P45379 | P62736 |  |
|  |  |  |  | Q13618 | P16066 |  |
|  |  |  |  | P05412 | P15408 |  |
|  |  |  |  | Q02763 | P02776 |  |
|  |  |  |  | P51681 | Q15797 |  |
|  |  |  |  | P51684 | P01138 |  |
|  |  |  |  | P02778 | P05121 |  |
|  |  |  |  | P07949 | P27487 |  |
|  |  |  |  | P04179 | O00585 |  |
|  |  |  |  | P42574 | P35869 |  |
|  |  |  |  | P08493 | Q16552 |  |
|  |  |  |  | P01303 | Q9Y5S8 |  |
|  |  |  |  | P11684 | Q13642 |  |
|  |  |  |  | P14210 | P10176 |  |
|  |  |  |  | P05093 | Q9UEF7 |  |
|  |  |  |  | P12644 | P20810 |  |
|  |  |  |  | P17931 | P10144 |  |
|  |  |  |  | P11712 | Q9P246 |  |
|  |  |  |  | Q9ULZ1 | P36222 |  |
|  |  |  |  | Q9Y210 | P26599 |  |
|  |  |  |  | O00238 | P14618 |  |
|  |  |  |  | P01011 | Q92908 |  |
|  |  |  |  | Q15796 | Q9NTG7 |  |
|  |  |  |  | P60484 | O15123 |  |
|  |  |  |  | P01133 | Q9HCE7 |  |
|  |  |  |  | P11226 | P21333 |  |
|  |  |  |  | P10826 | P25105 |  |
|  |  |  |  | P05113 | Q99758 |  |
|  |  |  |  | P12931 | P45983 |  |
|  |  |  |  | P09211 | P49238 |  |
|  |  |  |  | P27169 | P35408 |  |
|  |  |  |  | P02545 | P01100 |  |
|  |  |  |  | P15056 | P19474 |  |
|  |  |  |  | P60568 | P29965 |  |
|  |  |  |  | P22413 | P40337 |  |
|  |  |  |  | P09917 | P05412 |  |
|  |  |  |  | P35568 | P10646 |  |
|  |  |  |  | P17342 | P23560 |  |
|  |  |  |  | P04798 | P01034 |  |
|  |  |  |  | P48023 | P48681 |  |
|  |  |  |  | P21964 | P27815 |  |
|  |  |  |  | Q9HC29 | Q8NER1 |  |
|  |  |  |  | O95467 | Q16875 |  |
|  |  |  |  | P63092 | O43927 |  |
|  |  |  |  | P84996 | Q14511 |  |
|  |  |  |  | Q5JWF2 | P00746 |  |
|  |  |  |  | Q9H5I5 | P25116 |  |
|  |  |  |  | P36894 |  |  |
|  |  |  |  | Q9BUM1 |  |  |
|  |  |  |  | P06213 |  |  |
|  |  |  |  | P02749 |  |  |
|  |  |  |  | P14138 |  |  |
|  |  |  |  | Q15797 |  |  |
|  |  |  |  | P07099 |  |  |
|  |  |  |  | Q9UBU3 |  |  |
|  |  |  |  | O95436 |  |  |
|  |  |  |  | P61812 |  |  |
|  |  |  |  | P07492 |  |  |
|  |  |  |  | P49815 |  |  |
|  |  |  |  | P05089 |  |  |
|  |  |  |  | O75431 |  |  |
|  |  |  |  | P01024 |  |  |
|  |  |  |  | P28482 |  |  |
|  |  |  |  | P24298 |  |  |
|  |  |  |  | Q99967 |  |  |
|  |  |  |  | P39900 |  |  |
|  |  |  |  | P05091 |  |  |
|  |  |  |  | Q13976 |  |  |
|  |  |  |  | P08123 |  |  |
|  |  |  |  | P30411 |  |  |
|  |  |  |  | P00813 |  |  |
|  |  |  |  | P02675 |  |  |
|  |  |  |  | Q6PI48 |  |  |
|  |  |  |  | P02776 |  |  |
|  |  |  |  | P20800 |  |  |
|  |  |  |  | P13945 |  |  |
|  |  |  |  | P51671 |  |  |
|  |  |  |  | P49682 |  |  |
|  |  |  |  | P48357 |  |  |
|  |  |  |  | P29460 |  |  |
|  |  |  |  | P40337 |  |  |
|  |  |  |  | P26022 |  |  |
|  |  |  |  | Q07889 |  |  |
|  |  |  |  | Q9UMS0 |  |  |
|  |  |  |  | P30711 |  |  |
|  |  |  |  | Q9BX79 |  |  |
|  |  |  |  | P19838 |  |  |
|  |  |  |  | P14174 |  |  |
|  |  |  |  | P01034 |  |  |
|  |  |  |  | Q07869 |  |  |
|  |  |  |  | P55774 |  |  |
|  |  |  |  | P08473 |  |  |
|  |  |  |  | P01374 |  |  |
|  |  |  |  | P01920 |  |  |
|  |  |  |  | P16284 |  |  |
|  |  |  |  | P42345 |  |  |
|  |  |  |  | P28300 |  |  |
|  |  |  |  | P55017 |  |  |
|  |  |  |  | Q99814 |  |  |
|  |  |  |  | P13533 |  |  |
|  |  |  |  | Q16854 |  |  |
|  |  |  |  | P16410 |  |  |
|  |  |  |  | P41597 |  |  |
|  |  |  |  | P10635 |  |  |
|  |  |  |  | P01344 |  |  |
|  |  |  |  | P29965 |  |  |
|  |  |  |  | P16671 |  |  |
|  |  |  |  | Q92902 |  |  |
|  |  |  |  | P61769 |  |  |
|  |  |  |  | Q08499 |  |  |
|  |  |  |  | P69892 |  |  |
|  |  |  |  | P48061 |  |  |
|  |  |  |  | P29475 |  |  |
|  |  |  |  | P40225 |  |  |
|  |  |  |  | P01127 |  |  |
|  |  |  |  | Q99988 |  |  |
|  |  |  |  | P09429 |  |  |
|  |  |  |  | P68871 |  |  |
|  |  |  |  | P01236 |  |  |
|  |  |  |  | P24158 |  |  |
|  |  |  |  | P03372 |  |  |
|  |  |  |  | Q6N021 |  |  |
|  |  |  |  | P10600 |  |  |
|  |  |  |  | P02461 |  |  |
|  |  |  |  | P36222 |  |  |
|  |  |  |  | Q06330 |  |  |
|  |  |  |  | Q16678 |  |  |
|  |  |  |  | P01730 |  |  |
|  |  |  |  | P80188 |  |  |
|  |  |  |  | Q16585 |  |  |
|  |  |  |  | P25445 |  |  |
|  |  |  |  | P36382 |  |  |
|  |  |  |  | P20333 |  |  |
|  |  |  |  | P02753 |  |  |
|  |  |  |  | Q01668 |  |  |
|  |  |  |  | Q13568 |  |  |
|  |  |  |  | O00141 |  |  |
|  |  |  |  | P04839 |  |  |
|  |  |  |  | P14778 |  |  |
|  |  |  |  | P02775 |  |  |
|  |  |  |  | P07996 |  |  |
|  |  |  |  | P42224 |  |  |
|  |  |  |  | P07101 |  |  |
|  |  |  |  | P11215 |  |  |
|  |  |  |  | Q9UM47 |  |  |
|  |  |  |  | Q9UM00 |  |  |
|  |  |  |  | Q9NR61 |  |  |
|  |  |  |  | O15123 |  |  |
|  |  |  |  | O14521 |  |  |
|  |  |  |  | P21781 |  |  |
|  |  |  |  | Q4G0P3 |  |  |
|  |  |  |  | Q96PU5 |  |  |
|  |  |  |  | O95865 |  |  |
|  |  |  |  | P08F94 |  |  |
|  |  |  |  | P04035 |  |  |
|  |  |  |  | O43435 |  |  |
|  |  |  |  | P27361 |  |  |
|  |  |  |  | Q16539 |  |  |
|  |  |  |  | P41180 |  |  |
|  |  |  |  | P04440 |  |  |
|  |  |  |  | Q9Y5S8 |  |  |
|  |  |  |  | O95477 |  |  |
|  |  |  |  | Q92574 |  |  |
|  |  |  |  | P01189 |  |  |
|  |  |  |  | P23582 |  |  |
|  |  |  |  | P08684 |  |  |
|  |  |  |  | P41220 |  |  |
|  |  |  |  | P11150 |  |  |
|  |  |  |  | P00390 |  |  |
|  |  |  |  | P05413 |  |  |
|  |  |  |  | P23946 |  |  |
|  |  |  |  | O95180 |  |  |
|  |  |  |  | P09919 |  |  |
|  |  |  |  | Q04721 |  |  |
|  |  |  |  | P23219 |  |  |
|  |  |  |  | P02787 |  |  |
|  |  |  |  | Q9UBK2 |  |  |
|  |  |  |  | P34932 |  |  |
|  |  |  |  | P11362 |  |  |
|  |  |  |  | P00450 |  |  |
|  |  |  |  | P10721 |  |  |
|  |  |  |  | P20674 |  |  |
|  |  |  |  | P0DMV8 |  |  |
|  |  |  |  | P00395 |  |  |
|  |  |  |  | P35916 |  |  |
|  |  |  |  | P18075 |  |  |
|  |  |  |  | O60565 |  |  |
|  |  |  |  | P54802 |  |  |
|  |  |  |  | P00488 |  |  |
|  |  |  |  | Q9UM73 |  |  |
|  |  |  |  | P04049 |  |  |
|  |  |  |  | Q30201 |  |  |
|  |  |  |  | P08603 |  |  |
|  |  |  |  | Q9UMR3 |  |  |
|  |  |  |  | P19438 |  |  |
|  |  |  |  | P21912 |  |  |
|  |  |  |  | P02144 |  |  |
|  |  |  |  | Q8WW38 |  |  |
|  |  |  |  | P11597 |  |  |
|  |  |  |  | P43119 |  |  |
|  |  |  |  | P02766 |  |  |
|  |  |  |  | Q14654 |  |  |
|  |  |  |  | P16035 |  |  |
|  |  |  |  | P07911 |  |  |
|  |  |  |  | P39060 |  |  |
|  |  |  |  | P56706 |  |  |
|  |  |  |  | P00747 |  |  |
|  |  |  |  | P01185 |  |  |
|  |  |  |  | P61073 |  |  |
|  |  |  |  | P35414 |  |  |
|  |  |  |  | P17661 |  |  |
|  |  |  |  | P48995 |  |  |
|  |  |  |  | P48736 |  |  |
|  |  |  |  | O94760 |  |  |
|  |  |  |  | P28845 |  |  |
|  |  |  |  | P42336 |  |  |
|  |  |  |  | Q96EB6 |  |  |
|  |  |  |  | P08729 |  |  |
|  |  |  |  | Q2M1Z3 |  |  |
|  |  |  |  | P02774 |  |  |
|  |  |  |  | P12724 |  |  |
|  |  |  |  | P20366 |  |  |
|  |  |  |  | P09237 |  |  |
|  |  |  |  | P29033 |  |  |
|  |  |  |  | Q96P20 |  |  |
|  |  |  |  | P01589 |  |  |
|  |  |  |  | P19544 |  |  |
|  |  |  |  | P23560 |  |  |
|  |  |  |  | P08758 |  |  |
|  |  |  |  | P35749 |  |  |
|  |  |  |  | O95967 |  |  |
|  |  |  |  | Q9UQQ2 |  |  |
|  |  |  |  | P14672 |  |  |
|  |  |  |  | P08519 |  |  |
|  |  |  |  | O60706 |  |  |
|  |  |  |  | P21817 |  |  |
|  |  |  |  | P19440 |  |  |
|  |  |  |  | Q02750 |  |  |
|  |  |  |  | Q9GZV9 |  |  |
|  |  |  |  | P08183 |  |  |
|  |  |  |  | P12830 |  |  |
|  |  |  |  | P04439 |  |  |
|  |  |  |  | O00300 |  |  |
|  |  |  |  | P16066 |  |  |
|  |  |  |  | P08069 |  |  |
|  |  |  |  | P32297 |  |  |
|  |  |  |  | P78380 |  |  |
|  |  |  |  | Q9NPJ1 |  |  |
|  |  |  |  | Q15063 |  |  |
|  |  |  |  | P41595 |  |  |
|  |  |  |  | P01100 |  |  |
|  |  |  |  | P42771 |  |  |
|  |  |  |  | Q8N726 |  |  |
|  |  |  |  | P43490 |  |  |
|  |  |  |  | P18031 |  |  |
|  |  |  |  | P42772 |  |  |
|  |  |  |  | P34913 |  |  |
|  |  |  |  | P49279 |  |  |
|  |  |  |  | P25025 |  |  |
|  |  |  |  | P35612 |  |  |
|  |  |  |  | Q9UBX5 |  |  |
|  |  |  |  | P28906 |  |  |
|  |  |  |  | P28222 |  |  |
|  |  |  |  | P17752 |  |  |
|  |  |  |  | Q13093 |  |  |
|  |  |  |  | P24385 |  |  |
|  |  |  |  | Q03167 |  |  |
|  |  |  |  | P50148 |  |  |
|  |  |  |  | P25116 |  |  |
|  |  |  |  | P16234 |  |  |
|  |  |  |  | O95760 |  |  |
|  |  |  |  | Q9Y2R2 |  |  |
|  |  |  |  | O14788 |  |  |
|  |  |  |  | Q9C0B1 |  |  |
|  |  |  |  | O00443 |  |  |
|  |  |  |  | Q9UEF7 |  |  |
|  |  |  |  | Q96HP0 |  |  |
|  |  |  |  | Q07325 |  |  |
|  |  |  |  | O14791 |  |  |
|  |  |  |  | P98088 |  |  |
|  |  |  |  | Q13464 |  |  |
|  |  |  |  | Q9NZD4 |  |  |
|  |  |  |  | P05783 |  |  |
|  |  |  |  | P53667 |  |  |
|  |  |  |  | P33151 |  |  |
|  |  |  |  | P35579 |  |  |
|  |  |  |  | Q15746 |  |  |
|  |  |  |  | P12883 |  |  |
|  |  |  |  | Q9NRR6 |  |  |
|  |  |  |  | O75116 |  |  |
|  |  |  |  | Q07075 |  |  |
|  |  |  |  | P08709 |  |  |
|  |  |  |  | P05106 |  |  |
|  |  |  |  | P21728 |  |  |
|  |  |  |  | P09172 |  |  |
|  |  |  |  | Q01995 |  |  |
|  |  |  |  | P40238 |  |  |
|  |  |  |  | P00451 |  |  |
|  |  |  |  | P09104 |  |  |
|  |  |  |  | Q92736 |  |  |
|  |  |  |  | P09619 |  |  |
|  |  |  |  | P63000 |  |  |
|  |  |  |  | P35520 |  |  |
|  |  |  |  | P00403 |  |  |
|  |  |  |  | P38936 |  |  |
|  |  |  |  | P04278 |  |  |
|  |  |  |  | Q02928 |  |  |
|  |  |  |  | Q9BSI4 |  |  |
|  |  |  |  | P16220 |  |  |
|  |  |  |  | Q92731 |  |  |
|  |  |  |  | Q9UPY3 |  |  |
|  |  |  |  | P08571 |  |  |
|  |  |  |  | P00742 |  |  |
|  |  |  |  | Q16236 |  |  |
|  |  |  |  | P01112 |  |  |
|  |  |  |  | P51801 |  |  |
|  |  |  |  | P14151 |  |  |
|  |  |  |  | Q13507 |  |  |
|  |  |  |  | Q99972 |  |  |
|  |  |  |  | Q96QV1 |  |  |
|  |  |  |  | Q9NZ08 |  |  |
|  |  |  |  | P01111 |  |  |
|  |  |  |  | P05023 |  |  |
|  |  |  |  | Q14767 |  |  |
|  |  |  |  | P51589 |  |  |
|  |  |  |  | P98160 |  |  |

**Table S3.** Calculation of various parameters of the network.

| **Genes** | **AverageShortestPathLength** | **BetweennessCentrality** | **ClosenessCentrality** | **ClusteringCoefficient** | **Degree** |
| --- | --- | --- | --- | --- | --- |
| STAT3 | 1.280374 | 0.010295 | 0.781022 | 0.541012 | 149 |
| FOS | 1.280374 | 0.008081 | 0.781022 | 0.592618 | 120 |
| MAPK1 | 1.448598 | 0.007145 | 0.690323 | 0.50789 | 119 |
| CAV1 | 1.308411 | 0.010164 | 0.764286 | 0.533876 | 114 |
| AKT1 | 1.439252 | 0.007068 | 0.694805 | 0.528249 | 110 |
| TP53 | 1.514019 | 0.005285 | 0.660494 | 0.538462 | 107 |
| EP300 | 1.46729 | 0.005996 | 0.681529 | 0.540727 | 106 |
| BMP2 | 1.327103 | 0.00829 | 0.753521 | 0.572379 | 104 |
| TGFBR2 | 1.373832 | 0.007229 | 0.727891 | 0.567616 | 103 |
| STAT1 | 1.560748 | 0.002842 | 0.640719 | 0.607771 | 99 |
| JAK2 | 1.457944 | 0.005626 | 0.685897 | 0.558379 | 96 |
| PPARG | 1.364486 | 0.009516 | 0.732877 | 0.525022 | 96 |
| IL6 | 1.429907 | 0.004513 | 0.699346 | 0.643169 | 94 |
| FLNA | 1.373832 | 0.00771 | 0.727891 | 0.559928 | 92 |
| TGFBR1 | 1.46729 | 0.005876 | 0.681529 | 0.536967 | 91 |
| RAF1 | 1.485981 | 0.005564 | 0.672956 | 0.53468 | 88 |
| COL1A2 | 1.448598 | 0.00686 | 0.690323 | 0.558738 | 88 |
| IL1B | 1.457944 | 0.004202 | 0.685897 | 0.643678 | 86 |
| IL12A | 1.457944 | 0.006091 | 0.685897 | 0.565638 | 84 |
| SOS1 | 1.495327 | 0.00599 | 0.66875 | 0.512928 | 83 |
| THBD | 1.392523 | 0.006047 | 0.718121 | 0.605769 | 82 |
| GJA1 | 1.401869 | 0.0072 | 0.713333 | 0.571429 | 82 |
| CCND1 | 1.476636 | 0.004936 | 0.677215 | 0.566234 | 81 |
| KRAS | 1.607477 | 0.002735 | 0.622093 | 0.576074 | 79 |
| NOTCH1 | 1.46729 | 0.006236 | 0.681529 | 0.547619 | 78 |
| HLA-B | 1.476636 | 0.005003 | 0.677215 | 0.601299 | 75 |
| HMOX1 | 1.401869 | 0.007956 | 0.713333 | 0.582837 | 75 |
| SMAD4 | 1.598131 | 0.003058 | 0.625731 | 0.565891 | 75 |
| TGFB1 | 1.476636 | 0.005647 | 0.677215 | 0.568831 | 75 |
| PTEN | 1.514019 | 0.004036 | 0.660494 | 0.573152 | 75 |
| ADRB2 | 1.373832 | 0.007694 | 0.727891 | 0.581185 | 75 |
| IL10 | 1.476636 | 0.004973 | 0.677215 | 0.612338 | 72 |
| HLA-DRB1 | 1.439252 | 0.004957 | 0.694805 | 0.607345 | 71 |
| CDKN2A | 1.579439 | 0.003874 | 0.633136 | 0.524242 | 68 |
| MMP1 | 1.46729 | 0.004859 | 0.681529 | 0.617168 | 67 |
| COL3A1 | 1.588785 | 0.002389 | 0.629412 | 0.663848 | 66 |
| IL1A | 1.551402 | 0.003112 | 0.644578 | 0.639184 | 65 |
| IL12B | 1.598131 | 0.001912 | 0.625731 | 0.697674 | 62 |
| BRAF | 1.700935 | 0.001591 | 0.587912 | 0.618952 | 62 |
| SPP1 | 1.542056 | 0.00325 | 0.648485 | 0.610544 | 59 |
| FGFR1 | 1.598131 | 0.004734 | 0.625731 | 0.516058 | 59 |
| WT1 | 1.560748 | 0.005665 | 0.640719 | 0.490287 | 58 |
| IKBKG | 1.626168 | 0.004107 | 0.614943 | 0.50641 | 57 |
| VHL | 1.64486 | 0.003197 | 0.607955 | 0.523471 | 56 |
| BMPR2 | 1.626168 | 0.002526 | 0.614943 | 0.573077 | 56 |
| CCR6 | 1.570093 | 0.004137 | 0.636905 | 0.525604 | 54 |
| SLC2A10 | 1.551402 | 0.005398 | 0.644578 | 0.518617 | 50 |
| CYP1A1 | 1.616822 | 0.004432 | 0.618497 | 0.509756 | 45 |
| FMR1 | 1.598131 | 0.003245 | 0.625731 | 0.533776 | 45 |
| TNFSF11 | 1.682243 | 0.002042 | 0.594444 | 0.611408 | 39 |
| NOS3 | 1.728972 | 0.001654 | 0.578378 | 0.522167 | 39 |
| IGF2 | 1.813084 | 4.38E-04 | 0.551546 | 0.657895 | 27 |
| PRTN3 | 1.775701 | 0.001353 | 0.563158 | 0.514493 | 24 |
| COMT | 1.785047 | 6.86E-04 | 0.560209 | 0.600791 | 24 |
