# Supplementary material for: Quercetin inhibits angiotensin II-induced vascular smooth muscle cell proliferation and activation of JAK2/STAT3 pathway: A target based networking pharmacology approach
Source: Front Pharmacol. 2022 Oct 17;13:1002363. doi: 10.3389/fphar.2022.1002363 (PMC9618806; doi:10.3389/fphar.2022.1002363)
Supplement: Supplementary file 1 [file DataSheet2.ZIP › Supplemetary files/WB raw data.pptx]

## Slide 1
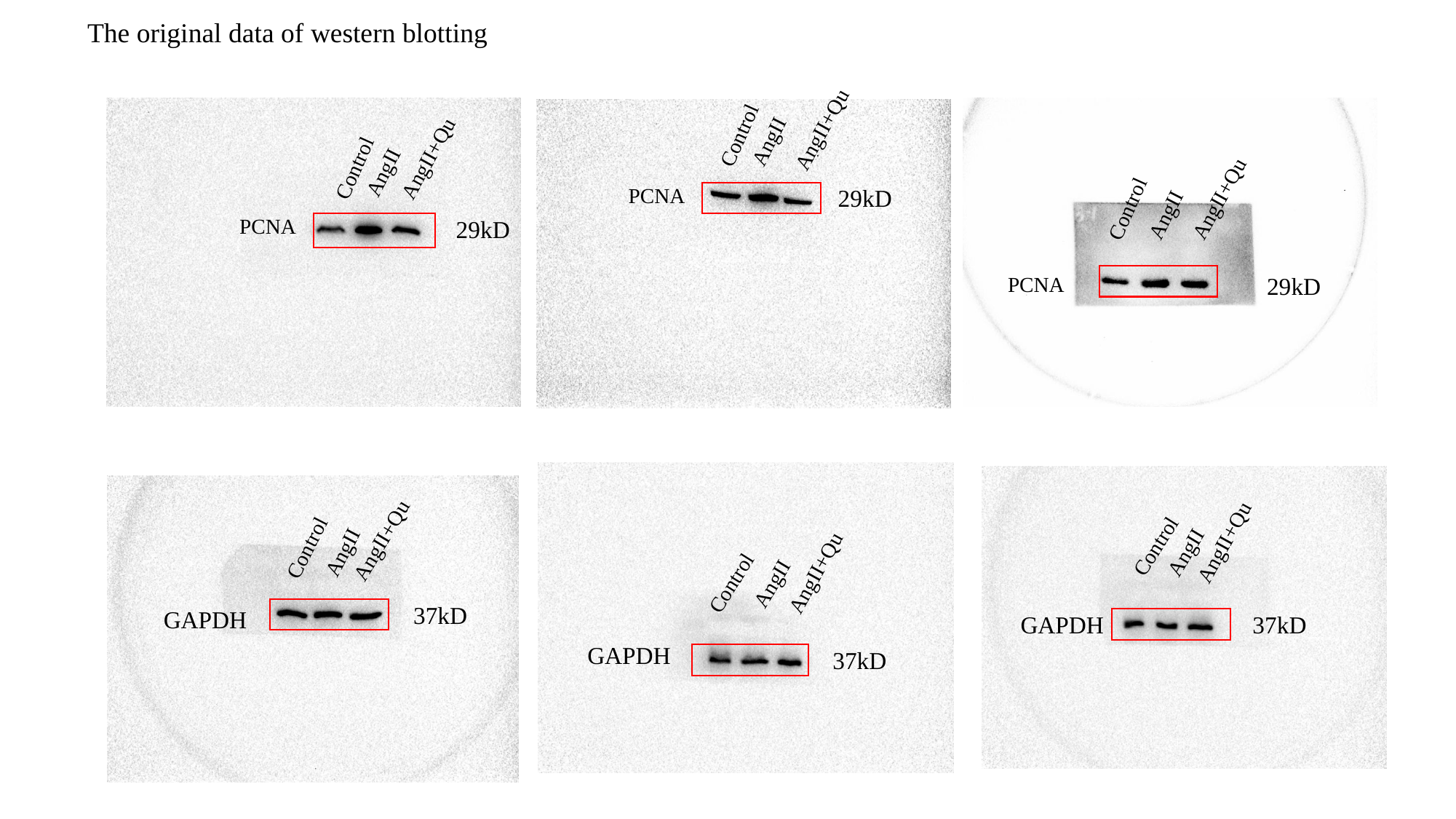

The original data of western blotting
AngII
Control
AngII+Qu
AngII
AngII+Qu
Control
PCNA
AngII
29kD
Control
AngII+Qu
PCNA
29kD
29kD
PCNA
AngII+Qu
AngII+Qu
AngII
AngII
Control
Control
AngII
Control
AngII+Qu
37kD
GAPDH
GAPDH
37kD
GAPDH
37kD

## Slide 2
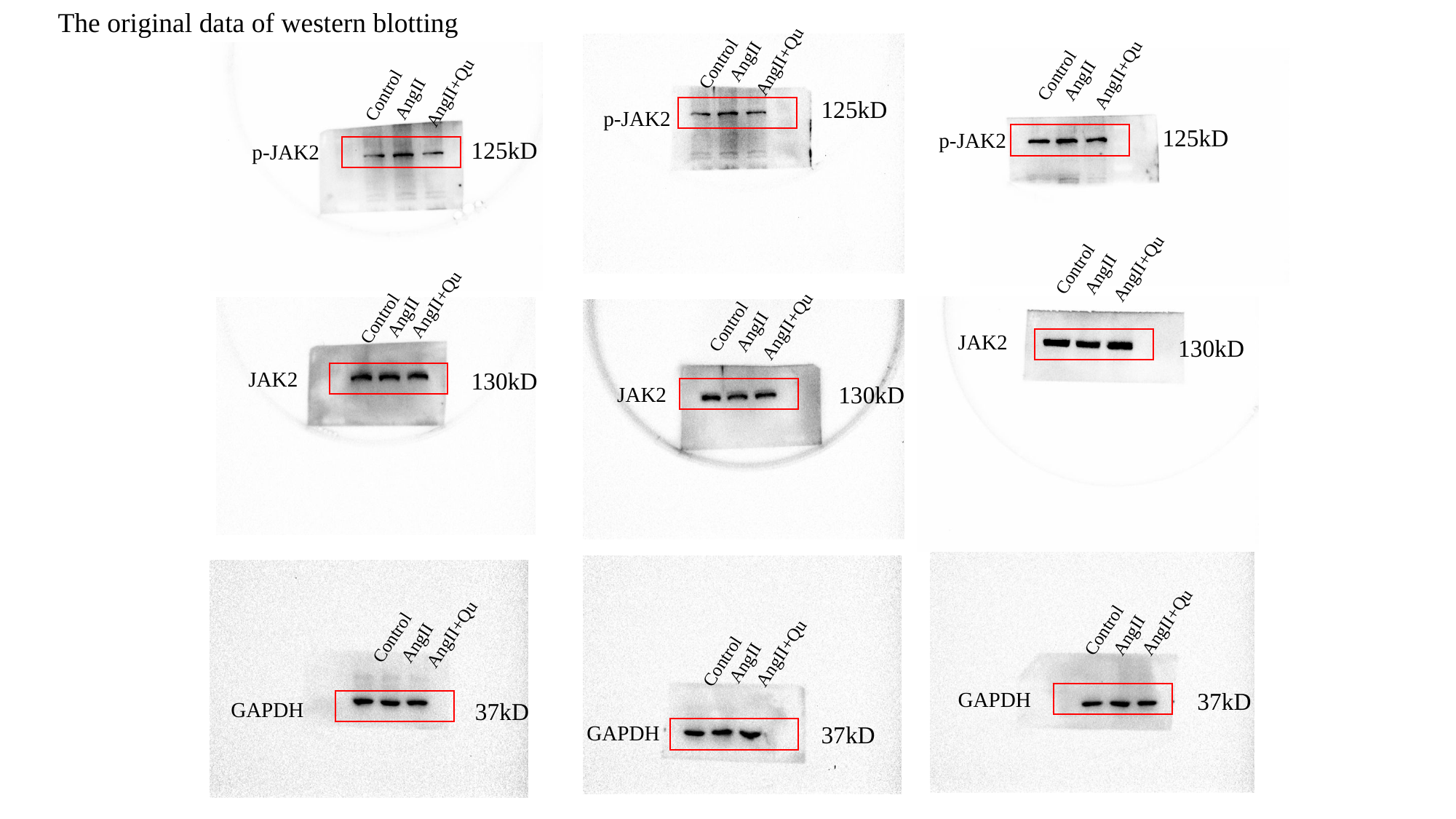

The original data of western blotting
AngII
Control
AngII
Control
AngII+Qu
AngII+Qu
AngII
Control
AngII+Qu
125kD
p-JAK2
125kD
p-JAK2
125kD
p-JAK2
AngII
Control
AngII+Qu
AngII
AngII+Qu
Control
Control
AngII
AngII+Qu
JAK2
130kD
JAK2
130kD
130kD
JAK2
AngII+Qu
AngII
Control
Control
AngII
AngII+Qu
AngII
Control
AngII+Qu
GAPDH
37kD
37kD
GAPDH
GAPDH
37kD

## Slide 3
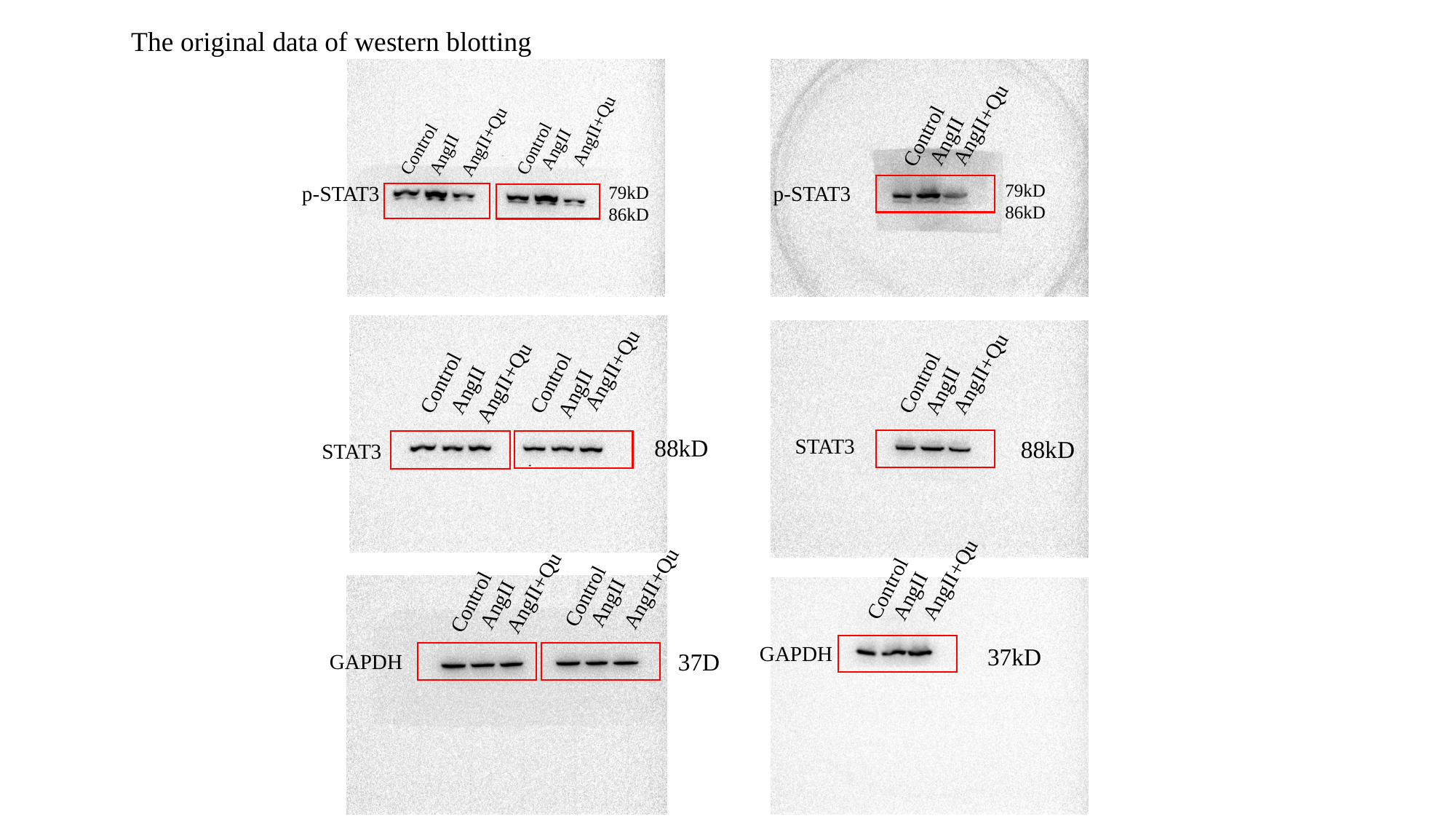

The original data of western blotting
AngII
AngII+Qu
Control
AngII+Qu
AngII
Control
AngII
Control
AngII+Qu
79kD
86kD
p-STAT3
p-STAT3
79kD
86kD
AngII+Qu
Control
Control
Control
AngII
AngII+Qu
AngII
AngII+Qu
AngII
88kD
STAT3
88kD
STAT3
AngII+Qu
Control
AngII
AngII
Control
AngII+Qu
AngII
Control
AngII+Qu
GAPDH
37kD
37D
GAPDH
